# Blinded sample size re-estimation in a comparative diagnostic accuracy study

**DOI:** 10.1186/s12874-022-01564-2

**Published:** 2022-04-19

**Authors:** Maria Stark, Mailin Hesse, Werner Brannath, Antonia Zapf

**Affiliations:** 1 University Medical Center Hamburg-Eppendorf, Institute of Medical Biometry and Epidemiology, Martinistr. 52, 20246 Hamburg, Germany; 2 Abbott GmbH, Wiesbaden, Germany; 3 University of Bremen, Institute of Statistics, Bremen, Germany

**Keywords:** Adaptive design, Co-primary endpoints, Sensitivity, Specificity, Unpaired design, Paired design

## Abstract

**Background:**

The sample size calculation in a confirmatory diagnostic accuracy study is performed for co-primary endpoints because sensitivity and specificity are considered simultaneously. The initial sample size calculation in an unpaired and paired diagnostic study is based on assumptions about, among others, the prevalence of the disease and, in the paired design, the proportion of discordant test results between the experimental and the comparator test. The choice of the power for the individual endpoints impacts the sample size and overall power. Uncertain assumptions about the nuisance parameters can additionally affect the sample size.

**Methods:**

We develop an optimal sample size calculation considering co-primary endpoints to avoid an overpowered study in the unpaired and paired design. To adjust assumptions about the nuisance parameters during the study period, we introduce a blinded adaptive design for sample size re-estimation for the unpaired and the paired study design. A simulation study compares the adaptive design to the fixed design. For the paired design, the new approach is compared to an existing approach using an example study.

**Results:**

Due to blinding, the adaptive design does not inflate type I error rates. The adaptive design reaches the target power and re-estimates nuisance parameters without any relevant bias. Compared to the existing approach, the proposed methods lead to a smaller sample size.

**Conclusions:**

We recommend the application of the optimal sample size calculation and a blinded adaptive design in a confirmatory diagnostic accuracy study. They compensate inefficiencies of the sample size calculation and support to reach the study aim.

**Supplementary Information:**

The online version contains supplementary material available at 10.1186/s12874-022-01564-2.

## Background

**Fig. 1:**
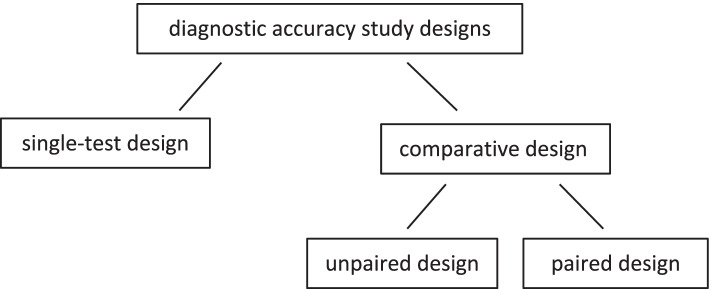
Study designs of a confirmatory diagnostic accuracy trial

In a diagnostic accuracy trial the experimental test is compared to the reference standard, which defines the true disease status. Either the evaluation is limited to the comparison with the reference standard (single-test design) or another test is considered in addition (comparative design) [1]. The present article puts the focus on comparative study designs in which the experimental test is compared to an already evaluated comparator test. In the unpaired design, either the experimental test or the comparator test is assigned randomly to study participants in addition to the reference standard [2]. In contrast, in the paired design, participants undergo all three diagnostic procedures [3]. Due to the within-subject comparison of the diagnostic tests in the paired design, the variability of the study results will be diminished [4]. For this reason, the paired design is preferred to the unpaired design if technically feasible and ethically justifiable [4]. Hence, the focus of this article is especially on the paired design. Figure 1 gives an overview about the different designs.

Independent of the chosen study design, sensitivity and specificity are used as co-primary endpoints in a confirmatory diagnostic accuracy trial [4, 5]. Both endpoints are combined via a joint hypothesis which is evaluated by the Intersection-Union Test [6, 7]. In this context, Stark et al. [8] developed an approach to calculate the sample size considering the prevalence. The advantage of this optimal sample size calculation is to avoid an overpowered study as it is often the case with the conventional approach. We will extend this approach to the unpaired and paired comparative study design. Hereby, the study might either aim to show superiority, non-inferiority or a combination of both regarding the co-primary endpoints.

To adjust the sample size during the course of the study, an adaptive design can be applied. Zapf et al. [9] reveal that adaptive designs including group-sequential designs are hardly developed and rarely applied in diagnostic studies. Stark et al. [8] introduce a blinded adaptive design for sample size re-estimation in the single-test design. Focusing on comparative study designs, Mazumdar et al. [10] propose a group-sequential design, but restricted to the area under the receiver operating characteristic curve as endpoint. McCray et al. [11] developed a blinded sample size re-estimation procedure in the paired study design regarding sensitivity and specificity. Their approach is based on the re-estimation of the proportion of concordant test results and the prevalence. To further develop the approaches of McCray et al. [11] and Stark et al. [8], we transfer the blinded adaptive design in the single-test design using the optimal sample size calculation to both comparative study designs. Hence, novel aspects in the present work are first, the development of the optimal sample size calculation in the unpaired as well as paired design aiming to show superiority, non-inferiority or a combination of both regarding the co-primary endpoints and second, the implementation of a blinded-sample size re-estimation procedure in the unpaired and paired design based on the optimal sample size calculation.

The present article is structured the following way: at first, we introduce the optimal sample size calculation in the unpaired and paired study design aiming to show superiority, non-inferiority or a combination of both. Second, we describe the procedure of the blinded sample size re-estimation in the unpaired and paired study design. Third, we compare the blinded adaptive design in a paired trial to the approach of McCray et al. [11] using an exemplary trial. Then, we present the results of a simulation study investigating the blinded adaptive design compared to a fixed design in an unpaired and paired study. Finally, we discuss the results and offer a conclusion.

## Methods

### Sample size calculation in a comparative diagnostic study

In this section, we introduce the optimal sample size calculation for a comparative diagnostic study, which is already developed by Stark et al. [8] for the single-test design. In a comparative diagnostic study, sensitivity and specificity of the experimental test can be tested for superiority, non-inferiority or the combination of superiority and non-inferiority against the comparator test. For the motivation and application of the optimal sample size calculation, we focus on the paired design testing for superiority regarding both endpoints because the paired design is the more relevant design in comparative studies [4]. However, the advantages of the optimal sample size calculation are also valid in the unpaired design. Furthermore, we provide formulas for the optimal approach in the unpaired and paired design.

In confirmatory diagnostic studies, sensitivity and specificity are combined as co-primary endpoints via the Intersection-Union test [8]. The null hypothesis of the Intersection-Union-Test is the union of the individual null hypothesis regarding sensitivity and the individual null hypothesis regarding specificity [6]. The overall power of this Intersection-Union test is calculated by the product of the power of each individual hypothesis. To show superiority of the experimental test regarding sensitivity and specificity against the comparator test, the global null hypothesis ${H}_{0_{\mathrm{global}}}$ for equality is given by:

1 $${\displaystyle \begin{array}{c}{\mathrm{H}}_{0_{\mathrm{Se}}}:\mathrm{S}{\mathrm{e}}_{\mathrm{E}}=\mathrm{S}{\mathrm{e}}_{\mathrm{C}}\ \mathrm{and}\ {\mathrm{H}}_{0_{\mathrm{Sp}}}:\mathrm{S}{\mathrm{p}}_{\mathrm{E}}=\mathrm{S}{\mathrm{p}}_{\mathrm{C}}\\ {}{\mathrm{H}}_{0_{\mathrm{global}}}={\mathrm{H}}_{0_{\mathrm{Se}}}\cup {\mathrm{H}}_{0_{\mathrm{Sp}}}\end{array}}$$

Se_E_ and Sp_E_ denote the sensitivity and specificity of the experimental test. Se_C_ and Sp_C_ represent the sensitivity and specificity of the comparator test. ${\mathrm{H}}_{0_{\mathrm{global}}}$ is only rejected if both ${\mathrm{H}}_{0_{\mathrm{Se}}}$ and ${\mathrm{H}}_{0_{\mathrm{Sp}}}$ are rejected simultaneously. Superiority of the experimental test regarding sensitivity and specificity against the comparator test can be concluded from point estimates and *p*-values or confidence intervals. Sensitivity and specificity represent the success probabilities of a binomial distribution which follow an asymptotic normality in the case of a large sample [12]. For the analysis based on confidence intervals, we propose to use approximate 100 · (1 − *α*)% confidence intervals for the difference of two proportions.

### Conventional sample size calculation

To motivate the advantage of the optimal sample size calculation, we show the problems related to the procedure of the conventional sample size calculation in a confirmatory diagnostic study in the context of the paired design.

The conventional sample size calculation consists of three steps: calculate the needed number of diseased and non-diseased individuals, refer these numbers to the prevalence to receive numbers needed to show sensitivity and specificity and, choose the maximum to determine the final sample size [13–15].

We now perform these three steps for a paired diagnostic study mentioned in McCray et al. [11]. The example study compares the experimental combination of Positron Emission Tomography (PET) and computed tomography (CT) against CT alone to diagnose pancreatic cancer. The goal is to show superiority of the experimental test against the comparator test. The biopsy defines the true disease status. Table 1 shows the assumptions for sample size calculation used in this example. The disease prevalence *π* represents the proportion of diseased individuals on all individuals. Parameters *ψ*_D_ and *ψ*_ND_ denote the proportion of discordant test results in the diseased and non-diseased population, hence those proportions in which both diagnostic tests lead to different test results. The conventional approach plans the sample size for each endpoint with a power of 90% which theoretically leads in the product to an overall target power of approximately 80%. The significance level *α* is set to 5% per endpoint. The 1 − *α*/2 and 1 − *β* quantile of the standard normal distribution is denoted by *z*_1 − *α*/2_ and *z*_1 − *β*_. The individual steps are as follows:

1. Sample size of diseased individuals based on the formula of Miettinen et al. [16]:
  $${n}_{\mathrm{D}}=\frac{{\left({z}_{1-\alpha /2}\cdot {\psi}_{\mathrm{D}}+{z}_{1-{\beta}_{\mathrm{Se}}}\sqrt{\psi_{\mathrm{D}}^2-\frac{1}{4}{\left(\mathrm{S}{\mathrm{e}}_{\mathrm{C}}-\mathrm{S}{\mathrm{e}}_{\mathrm{E}}\right)}^2\left(3+{\psi}_{\mathrm{D}}\right)}\right)}^2}{\psi_{\mathrm{D}}{\left(\mathrm{S}{\mathrm{e}}_{\mathrm{C}}-\mathrm{S}{\mathrm{e}}_{\mathrm{E}}\right)}^2}=74$$
  Sample size of non-diseased individuals:
  $${n}_{\mathrm{ND}}=\frac{{\left({z}_{1-\alpha /2}\cdot {\psi}_{\mathrm{ND}}+{z}_{1-{\beta}_{\mathrm{Sp}}}\sqrt{\psi_{\mathrm{ND}}^2-\frac{1}{4}{\left(\mathrm{S}{\mathrm{p}}_{\mathrm{C}}-\mathrm{S}{\mathrm{p}}_{\mathrm{E}}\right)}^2\left(3+{\psi}_{\mathrm{ND}}\right)}\right)}^2}{\psi_{\mathrm{ND}}{\left(\mathrm{S}{\mathrm{p}}_{\mathrm{C}}-\mathrm{S}{\mathrm{p}}_{\mathrm{E}}\right)}^2}=47$$
2. Total sample size including at least *n*_Se_ diseased individuals:
  $${N}_{\mathrm{Se}}=\frac{n_{\mathrm{Se}}}{\pi }=\frac{74}{0.47}=157$$
  Total sample size including at least *n*_Sp_ non-diseased individuals:
  $${N}_{\mathrm{Sp}}=\frac{n_{\mathrm{Sp}}}{1-\pi }=\frac{47}{1-0.47}=88$$
3. $$N=\max \left({N}_{\mathrm{Se}},{N}_{\mathrm{Sp}}\right)=157$$

**Table 1:** Assumptions of the paired diagnostic accuracy trial for the comparison of the experimental Positron Emission Tomography (PET) combined with the computed tomography (CT) against the comparator test PET

General input parameters: Significance level per endpoint: $\boldsymbol{\alpha} =\mathbf{0.05}\ \left(\mathsf{two}-\mathsf{sided}\right),$ Overall Power: Power_**overall**_ ***=*** 1 ***− β***_**overall**_ ***=*** 0.8 Power per endpoint: Power_**Se**_ ***=*** Power_**Sp**_ ***=*** 1 ***− β***_**Se**_ ***=*** 1 ***− β***_**Sp**_ ***=*** 0.9			
Prevalence: ***π =*** 0.47	Comparator test (CT)	Experimental test (PET/CT)	Proportion of discordant test results
Diseased population	Se_C_ = 0.81	Se_E_ = 0.90	*ψ*_D_ = 0.09
Non-diseased population	Sp_C_ = 0.66	Sp_E_ = 0.80	*ψ*_ND_ = 0.14

The study recruits more individuals than would be necessary to show the specificity because the sensitivity determines the final sample size in this scenario. This can result in an overpowered study. If the prevalence was smaller, the difference between *N*_Se_ and *N*_Sp_ would be even larger. Vice versa, if the prevalence was larger, *N*_Sp_ would determine the final sample size. These discrepancies between the sample sizes of both endpoints can result in an overpowered study. To face this problem, we propose the optimal sample size calculation explained in the next section.

### Optimal sample size calculation

At first, we present the general idea of the optimal sample size calculation. Then, we expand the optimal sample size calculation in the single-test design developed by Stark et al. [8] to an unpaired and paired study. Furthermore, we provide formulas testing for superiority regarding both endpoints in the unpaired and paired design. In additional materials, we show hypotheses and sample size formulas testing for non-inferiority or combinations of superiority and non-inferiority [see Additional file 1]. Furthermore, we offer R-Code for the optimal sample size calculation considering superiority in both endpoints in additional materials [see Additional file 2].

The general idea behind the optimal sample size calculation consists of the individual splitting of the overall power (Power_overall_) to both endpoints, so that *N*_Se_ and *N*_Sp_ are equal. In this case, we won’t need to select a maximum from both sample sizes. Consequently, the final sample size is the smallest representative sample which allows to reach the desired overall power. We calculate the final sample size with the following equation in which the symbol “ $\overset{!}{=}$ ” denotes that terms on both sides must be equal:

2 $${N}_{\mathrm{Se}}\overset{!}{=}{N}_{\mathrm{Sp}}$$

3 $$\frac{n_{\mathrm{Se}}}{\pi}\overset{!}{=}\frac{n_{\mathrm{Sp}}}{1-\pi }$$

Under the condition:

4 $${\mathrm{Power}}_{\mathrm{Se}}\cdot \mathrm{Powe}{\mathrm{r}}_{\mathrm{Sp}}={\mathrm{Power}}_{\mathrm{overall}}$$

5 $$\left(1-{\beta}_{\mathrm{Se}}\right)\cdot \left(1-{\beta}_{\mathrm{Sp}}\right)={\mathrm{Power}}_{\mathrm{overall}}$$

6 $${\beta}_{\mathrm{Sp}}=\frac{1-{\beta}_{\mathrm{Se}}-\mathrm{Powe}{\mathrm{r}}_{\mathrm{overall}}}{1-{\beta}_{\mathrm{Se}}}=1-\frac{\mathrm{Powe}{\mathrm{r}}_{\mathrm{overall}}}{1-{\beta}_{\mathrm{Se}}}$$

In the following subsections, we plug the condition into the sample size calculation; noting that the resulting equations cannot be solved analytically respect to *β*_Se_.

### Unpaired design

In the unpaired design, the optimal sample size calculation uses the formula for the comparison of two independent proportions following Zhou et al. [1]:

$$\frac{{\left({z}_{\alpha /2}\sqrt{V_0\left({\mathrm{Se}}_{\mathrm{C}}-{\mathrm{Se}}_{\mathrm{E}}\right)}+{z}_{\beta_{\mathrm{Se}}}\sqrt{V_A\left({\mathrm{Se}}_{\mathrm{C}}-{\mathrm{Se}}_{\mathrm{E}}\right)\ }\right)}^2}{{\left({\mathrm{Se}}_{\mathrm{C}}-{\mathrm{Se}}_{\mathrm{E}}\right)}^2\cdot \pi}\overset{!}{=}$$

7 $$\frac{{\left({z}_{\alpha /2}\sqrt{V_0\left({\mathrm{Sp}}_{\mathrm{C}}-{\mathrm{Sp}}_{\mathrm{E}}\right)}+{z}_{\frac{1-{\beta}_{\mathrm{Se}}-{\mathrm{Power}}_{\mathrm{overall}}}{1-{\beta}_{\mathrm{Se}}}}\sqrt{V_A\left({\mathrm{Sp}}_{\mathrm{C}}-{\mathrm{Sp}}_{\mathrm{E}}\right)\ }\right)}^2}{{\left({\mathrm{Sp}}_{\mathrm{C}}-{\mathrm{Sp}}_{\mathrm{E}}\right)}^2\cdot \left(1-\pi \right)}$$

where *V*_0_(Se_C_ − Se_E_) and *V*_*A*_(Se_C_ − Se_E_) represent the variance of the difference between Se_C_ and Se_E_ under the null and alternative hypothesis, respectively. In the unpaired design, the variance *V*(Se_C_ − Se_E_) is defined as [1]:

8 $$V\left({\mathrm{Se}}_{\mathrm{C}}-{\mathrm{Se}}_{\mathrm{E}}\right)={\mathrm{Se}}_{\mathrm{C}}\cdot \left(1-{\mathrm{Se}}_{\mathrm{C}}\right)+{\mathrm{Se}}_{\mathrm{E}}\cdot \left(1-{\mathrm{Se}}_{\mathrm{E}}\right)$$

The variance *V*(Sp_C_ − Sp_E_) is calculated in analogy.

Although the sample size formula in Eq. (7) fits to the Wald confidence interval for the difference of two independent proportions, we propose to analyse the unpaired design with the two-sided 1- α Score confidence interval for the difference of two independent proportions [17]. The coverage probability of the Score confidence interval is closer to the nominal level compared to the Wald confidence interval [18–20].

### Paired design

In the paired design, the optimal sample size is based on the formula of Miettinen et al. [16]:

9 $${\displaystyle \begin{array}{c}\frac{{\left({z}_{1-\alpha /2}\cdot {\psi}_{\mathrm{D}}+{z}_{1-{\beta}_{\mathrm{Se}}}\sqrt{\psi_{\mathrm{D}}^2-\frac{1}{4}{\left(\mathrm{S}{\mathrm{e}}_{\mathrm{C}}-\mathrm{S}{\mathrm{e}}_{\mathrm{E}}\right)}^2\left(3+{\psi}_{\mathrm{D}}\right)}\right)}^2}{\psi_D{\left(\mathrm{S}{\mathrm{e}}_{\mathrm{C}}-\mathrm{S}{\mathrm{e}}_{\mathrm{E}}\right)}^2\pi}\overset{!}{=}\\ {}\frac{{\left({z}_{1-\alpha /2}\cdot {\psi}_{\mathrm{ND}}+{z}_{\frac{\mathrm{Powe}{\mathrm{r}}_{\mathrm{overall}}}{1-{\beta}_{\mathrm{Se}}}}\sqrt{\psi_{\mathrm{ND}}^2-\frac{1}{4}{\left(\mathrm{S}{\mathrm{p}}_{\mathrm{C}}-\mathrm{S}{\mathrm{p}}_{\mathrm{E}}\right)}^2\left(3+{\psi}_{\mathrm{ND}}\right)}\right)}^2}{\psi_{\mathrm{ND}}{\left(\mathrm{S}{\mathrm{p}}_{\mathrm{C}}-\mathrm{S}{\mathrm{p}}_{\mathrm{E}}\right)}^2\left(1-\pi \right)}\end{array}}$$

with *ψ*_D_ as the proportion of discordant test results in the diseased sample, which varies between [16, 21]:

10 $$\left|\mathrm{S}{\mathrm{e}}_{\mathrm{C}}-\mathrm{S}{\mathrm{e}}_{\mathrm{E}}\right|\le {\psi}_{\mathrm{D}}\le \mathrm{S}{\mathrm{e}}_{\mathrm{C}}+\mathrm{S}{\mathrm{e}}_{\mathrm{E}}-2\cdot \mathrm{S}{\mathrm{e}}_{\mathrm{C}}\cdot \mathrm{S}{\mathrm{e}}_{\mathrm{E}}$$

The interval of the proportion of discordant test results in the non-diseased sample *ψ*_ND_ is calculated in analogy by considering Sp_C_ and Sp_E_.

For two different proportions of discordant test results in the diseased (${\psi}_{{\mathrm{D}}_1},{\psi}_{{\mathrm{D}}_2}$) and non-diseased (${\psi}_{{\mathrm{ND}}_1},{\psi}_{{\mathrm{ND}}_2}$) population, the total sample size N(*ψ*_D_, *ψ*_ND_) in Eq. (9) is monotone increasing:

11 $${\displaystyle \begin{array}{c}{\psi}_{{\mathrm{D}}_1},{\psi}_{{\mathrm{D}}_2}\in \left[\left|\mathrm{S}{\mathrm{e}}_{\mathrm{C}}-\mathrm{S}{\mathrm{e}}_{\mathrm{E}}\right|;\mathrm{S}{\mathrm{e}}_{\mathrm{C}}+\mathrm{S}{\mathrm{e}}_{\mathrm{E}}-2\cdot \mathrm{S}{\mathrm{e}}_{\mathrm{C}}\cdot \mathrm{S}{\mathrm{e}}_{\mathrm{E}}\right]\ \mathrm{and}\\ {}{\psi}_{{\mathrm{ND}}_1},{\psi}_{{\mathrm{ND}}_2}\in \left[\left|\mathrm{S}{\mathrm{p}}_{\mathrm{C}}-\mathrm{S}{\mathrm{p}}_{\mathrm{E}}\right|;\mathrm{S}{\mathrm{p}}_{\mathrm{C}}+\mathrm{S}{\mathrm{p}}_{\mathrm{E}}-2\cdot \mathrm{S}{\mathrm{p}}_{\mathrm{C}}\cdot \mathrm{S}{\mathrm{p}}_{\mathrm{E}}\right]\\ {}{\psi}_{{\mathrm{D}}_1}\le {\psi}_{{\mathrm{D}}_2}\mathrm{and}\ {\psi}_{{\mathrm{ND}}_1}\le {\psi}_{{\mathrm{ND}}_2}\Rightarrow \mathrm{N}\left({\psi}_{{\mathrm{D}}_1},{\psi}_{{\mathrm{ND}}_1}\right)\le \mathrm{N}\left({\psi}_{{\mathrm{D}}_2},{\psi}_{{\mathrm{ND}}_2}\right)\end{array}}$$

In analogy to the unpaired design, we propose to analyse the paired design with the two-sided 1- α Tango’s asymptotic score confidence interval for the difference of two matched proportions [22, 23]. We recommend this based on the reason given above. Furthermore, the Wald confidence is not range preserving [24].

### Application of the optimal sample size calculation in the paired design

**Fig. 2:**
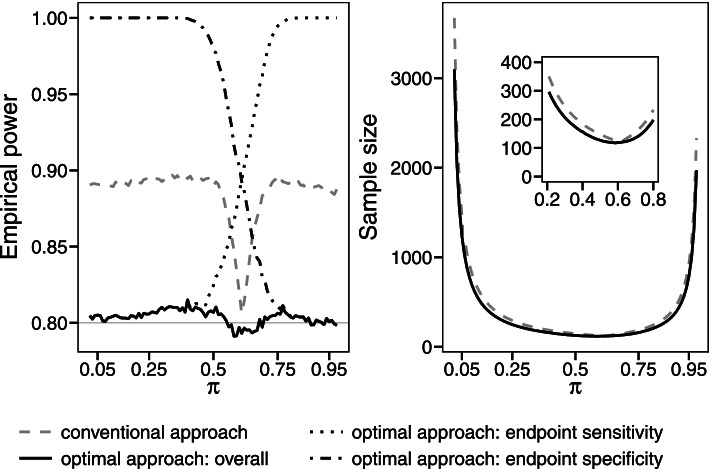
Empirical power and sample size of the conventional and optimal sample size calculation. Simulations are based on the example study given in Table 1 with a varying prevalence *π*. The figure considering the sample size contains an enlarged image section so that the differences between both approaches are highlighted

We apply the optimal sample size approach to the example study introduced in Table 1 and compare the results to those of the conventional approach. For this purpose, we simulate, based on 10,000 simulation runs, the empirical power of both approaches for a varying prevalence *π* and calculate the sample size. Figure 2 shows the results. In most cases, the conventional approach is highly overpowered due to the choice of the maximum sample size of both endpoints in the third step. If the prevalence is in the range between 0.5 and 0.75, the empirical power will be closer to the target power of 80%. The empirical power will be the closest to the target power, if the prevalence equals 0.6 as the discrepancy between *N*_Se_ and *N*_Sp_ is the smallest.

The optimal approach splits the overall power to both endpoints depending on the prevalence, so that the product of the empirical power of both endpoints comes close to the target power of 80%.

Considering the sample size, the optimal approach will lead to a smaller sample size than the conventional approach if the prevalence is unbalanced. Figure 2 contains an enlarged image section of the sample size so that the differences between both approaches are highlighted.

### Blinded sample size re-estimation

**Fig. 3:**
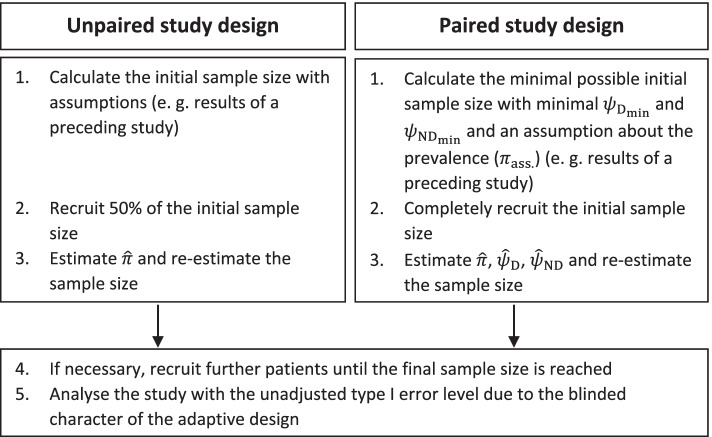
Procedure of the blinded adaptive design in an unpaired and paired diagnostic trial

The procedure of a blinded sample size adjustment based on the re-estimation of nuisance parameters basically follows five phases named by Stark et al. [8]. In Fig. 3, these five steps are explained in context of the unpaired and paired study design. The nuisance parameters re-estimated during the study are the prevalence and additionally proportions of discordant test results in the paired design. The main difference between the adaptive designs in the unpaired and paired study design consists of the sample size for the interim analysis. In the unpaired design, the prevalence is estimated based on 50% of the initially calculated sample size. In the paired design, both, the initial sample size and the sample size for the interim analysis equal the minimal sample size [11]. The minimal sample size is received with the minimal possible proportion of discordant test results in the diseased (${\psi}_{{\mathrm{D}}_{\mathrm{min}}}$) and non-diseased population (${\psi}_{{\mathrm{ND}}_{\mathrm{min}}}$). Assumptions about the sensitivity and the specificity of the comparator and experimental test determine the minimal possible proportion of discordant test results. Following Eq. (10), the minimal proportion of discordant test results are calculated with:

12 $${\displaystyle \begin{array}{c}{\psi}_{{\mathrm{D}}_{\mathrm{min}}}=\left|\mathrm{S}{\mathrm{e}}_{\mathrm{C}}-\mathrm{S}{\mathrm{e}}_{\mathrm{E}}\right|\\ {}{\psi}_{{\mathrm{ND}}_{\mathrm{min}}}=\left|\mathrm{S}{\mathrm{p}}_{\mathrm{C}}-\mathrm{S}{\mathrm{p}}_{\mathrm{E}}\right|\end{array}}$$

Furthermore, the calculation of the minimal sample size requires assumptions about the prevalence.

During interim analysis, the prevalence is estimated by the maximum likelihood estimator of a binomial proportion [25]:

13 $$\hat{\pi}=\frac{n_{\mathrm{D}}}{n}$$

The number of diseased individuals involved in the interim analysis is represented by *n*_D_, and the sample size used for interim analysis is denoted by *n*.

In analogy, the proportion of discordant test results is estimated by the maximum likelihood estimator of a multinomial distribution [26]:

14 $${\hat{\psi}}_{\mathrm{D}}=\frac{{n_{\mathrm{D}}}_{10}+{n_{\mathrm{D}}}_{01}}{n_{\mathrm{D}}}$$

15 $${\hat{\psi}}_{\mathrm{ND}}=\frac{{n_{\mathrm{ND}}}_{10}+{n_{\mathrm{ND}}}_{01}}{n_{\mathrm{ND}}}$$

Table 2 shows the parameters needed to re-estimate the proportions of discordant test results.

**Table 2:** Results in a paired diagnostic study

**Diseased** ***n***_**D**_			
		**Comparator Test**	
		**True** **Positive (TP**_**C**_**)**	**False** **Negative (FN**_**C**_**)**
**Experimental** **Test**	**True** **Positive (TP**_**E**_**)**	${n}_{{\mathrm{D}}_{11}}$	${n}_{{\mathrm{D}}_{10}}$
	**False** **Negative (FN**_**E**_**)**	${n}_{{\mathrm{D}}_{01}}$	${n}_{{\mathrm{D}}_{00}}$
**Non-diseased** ***n***_**ND**_			
		**Comparator Test**	
		**False** **Positive (FP**_**C**_**)**	**True** **Negative (TN**_**C**_**)**
**Experimental** **Test**	**False** **Positive (FP**_**E**_**)**	${n}_{{\mathrm{ND}}_{11}}$	${n}_{{\mathrm{ND}}_{10}}$
	**True** **Negative (TN**_**E**_**)**	${n}_{{\mathrm{ND}}_{01}}$	${n}_{{\mathrm{ND}}_{00}}$

The estimation of nuisance parameters represents a blinded adaptive design because the sensitivity and the specificity of the experimental test are not revealed. Hence, the type I error rate will not be inflated by definition.

## Results

### Application of the blinded sample size re-estimation in the example study

This section serves for illustration of the blinded sample size re-estimation in the paired study design. For this purpose, we compare the approach of McCray et al. [11] to the adaptive design procedure described in this article by taking up the example of a paired diagnostic accuracy study already introduced in Table 1. The main progress of our new approach compared to McCray et al. [11] is to implement the optimal sample size calculation. We reveal the advantage of the optimal sample size calculation in this context again.

Table 3 compares the theoretical aspects and the results of both adaptive design procedures. They differ in the definition of endpoints, hypothesis and in the way the sample size calculation is performed. McCray et al. [11] work with the quotient of sensitivities and the quotient of specificities of both diagnostic tests as endpoints. They use sample size formulas which rely on the true-positive-positive rate (TPPR) and true-negative-negative-rate (TNNR) [27]. TPPR denotes the proportion of test results in which both, the comparator test and the experimental test correctly diagnose a diseased individual. Vice versa, TNNR represents the proportion of test results in which both tests correctly return a negative test result. For initial sample size calculation, TPPR_max_ and TNNR_max_ are used, which represent the maximal possible TPPR and TNNR, respectively.

**Table 3:** Comparison of the blinded adaptive design procedure with McCray et al. [11]

		McCray et al. (2017)	Our approach
General information	**Endpoint**	$\frac{\mathrm{S}{\mathrm{e}}_{\mathrm{E}}}{\mathrm{S}{\mathrm{e}}_{\mathrm{C}}}$ and $\frac{\mathrm{S}{\mathrm{p}}_{\mathrm{E}}}{\mathrm{S}{\mathrm{p}}_{\mathrm{C}}}$	Se_E_ − Se_C_ and Sp_E_ − Sp_C_
	${\mathbf{H}}_{{\mathbf{0}}_{\mathbf{global}}}$	${\mathrm{H}}_{0_{\mathrm{S}\mathrm{e}}}:\frac{\mathrm{S}{\mathrm{e}}_{\mathrm{E}}}{{\mathrm{S}\mathrm{e}}_{\mathrm{C}}}=1\cup$ ${\mathrm{H}}_{0_{\mathrm{S}\mathrm{p}}}:\frac{\mathrm{S}{\mathrm{p}}_{\mathrm{E}}}{{\mathrm{S}\mathrm{p}}_{\mathrm{C}}}=1$	${\mathrm{H}}_{0_{\mathrm{Se}}}:\mathrm{S}{\mathrm{e}}_{\mathrm{E}}-{\mathrm{Se}}_{\mathrm{C}}=0\cup$ ${\mathrm{H}}_{0_{\mathrm{Sp}}}:\mathrm{S}{\mathrm{p}}_{\mathrm{E}}-{\mathrm{Sp}}_{\mathrm{C}}=0$
	**Sample size calculation**	Conventional approach *α* per endpoint: 0.05 (two-sided) Power per endpoint: 0.8	Optimal approach *α* per endpoint: 0.05 (two-sided) Overall power: 0.8
	**Parameter of dependency between both tests**	$\mathrm{TPPR}=\frac{{n_{\mathrm{D}}}_{11}}{n_{\mathrm{D}}}$ $\mathrm{TNNR}=\frac{{n_{\mathrm{ND}}}_{00}}{n_{\mathrm{ND}}}$	${\psi}_{\mathrm{D}}=\frac{{n_{\mathrm{D}}}_{10}+{n_{\mathrm{D}}}_{01}}{n_{\mathrm{D}}}$ ${\psi}_{\mathrm{ND}}=\frac{{n_{\mathrm{ND}}}_{10}+{n_{\mathrm{ND}}}_{01}}{n_{\mathrm{ND}}}$
Initial sample size calculation	**Size of internal pilot study**	TPPR_max_ and TNNR_max_ correspond to ${\psi}_{{\mathrm{D}}_{\mathrm{min}}}$ and ${\psi}_{{\mathrm{ND}}_{\mathrm{min}}}$	
	**Parameter of dependency between both tests for initial sample size calculation**	TPPR_max_ = Se_C_ = 0.81 TNNR_max_ = Sp_C_ = 0.66	${\psi}_{{\mathrm{D}}_{\mathrm{min}}}=\left|\mathrm{S}{\mathrm{e}}_{\mathrm{C}}-\mathrm{S}{\mathrm{e}}_{\mathrm{E}}\right|=0.09$ ${\psi}_{{\mathrm{ND}}_{\mathrm{min}}}=\left|\mathrm{S}{\mathrm{p}}_{\mathrm{C}}-\mathrm{S}{\mathrm{p}}_{\mathrm{E}}\right|=0.14$
	**Initial sample size, size of internal pilot study**	186	133
Sample size re-estimation	**Estimation of nuisance parameters**	$\hat{\pi}=0.44$ $\hat{TPPR}=0.80\mid$ $\hat{TNNR}=0.66$	$\hat{\pi}=0.44$ ${\hat{\psi}}_{\mathrm{D}}=0.11\mid$ ${\hat{\psi}}_{\mathrm{ND}}=0.14$
	**Re-estimated sample size**	242	200

McCray et al. [11] perform the sample size calculation based on the conventional three steps by planning the sample size calculation with a power of 80% per endpoint. This leads to a theoretical overall power of 64%.

In contrast to McCray et al. [11], our approach uses the optimal sample size calculation. It is based on sample size formulas considering the difference of sensitivities and the proportion of discordant test results in the diseased population or the difference of specificities of both tests and the proportion of discordant test results in the non-diseased population, respectively [1]. In contrast to McCray et al. [11], we choose the differences as endpoint measurement because the guideline on clinical evaluation of diagnostic agents suggests this [4]. Furthermore, we perform the optimal sample size calculation to reach an overall power of 80%.

Table 3 shows the initial sample size, the sample size for interim analysis and the re-estimated sample size of both adaptive design procedures. Due to the optimal approach, sample sizes resulting from our adaptive design are lower than those of McCray et al. [11]. The optimal sample size calculation avoids that one of both co-primary endpoints is overpowered which leads to smaller sample sizes.

The difference between both approaches regarding sample sizes will be even more extensive if the prevalence is unbalanced. A figure in additional materials, which depicts the simulated empirical overall power based on 10,000 simulations runs and the calculated sample size, illustrates this difference between both approaches for the initial sample size calculation based on ${\psi}_{{\mathrm{D}}_{\mathrm{min}}}$ and ${\psi}_{{\mathrm{ND}}_{\mathrm{min}}}$ by varying *π* [see Additional file 3]. This figure reveals that the approach of McCray et al [11]. is highly overpowered although they plan with a power of 80% per endpoint. This theoretically leads to a theoretical overall power of 64%. In this example, the dependence between both diagnostic tests is almost maximal because *ψ*_D_ and *ψ*_ND_ are almost minimal. In this case, the underlying assumptions of sample size formulas and confidence intervals are not valid [11]. Hence, the approach of McCray et al. [11] is highly overpowered.

In contrast, the optimal sample size calculation enables to reach an overall power of 80% independent of the prevalence.

### Simulation study

We perform a simulation study to evaluate type I error rates, statistical power, sample sizes and bias of the adaptive design based on re-estimated nuisance parameters in the unpaired and paired study design. We compare results of the adaptive design to those of the fixed design which gets by without re-estimation of the sample size. Table 4 shows the simulated scenarios testing for superiority in both endpoints. Based on the example of a paired diagnostic accuracy study used by McCray et al. [11], we choose one initial scenario. Starting from the initial scenario, we vary one parameter in each further scenario. That results in 15 scenarios in the unpaired design and 19 scenarios in the paired design, each simulated with 10,000 simulation runs. In analogy to these scenarios, we perform simulations testing for non-inferiority in both endpoints, or the combinations of superiority and non-inferiority, respectively. In this section, we focus on the results of those scenarios testing for superiority in both endpoints because the other results are comparable to them. For completeness, we make the remaining simulated scenarios and their results available in the online supplement materials [see Additional files 4 and 5].

**Table 4:** Simulated scenarios in the unpaired and paired study design testing for superiority in both endpoints. The proportion of discordant test results is only relevant in the paired design

	10,000 simulation runs per scenario	
Nominal significance level α per endpoint	0.05 (two-sided)	
Nominal overall target power	0.8	
	**Initial scenario**	**Variation of initial scenario**
Sensitivity comparator test Se_C_	0.8	0.6, 0.7
Specificity comparator test Sp_C_	0.7	0.6, 0.8
True prevalence ***π***_true_	0.2	0.4, 0.6, 0.8
Assumed prevalence ***π***_**ass.**_	*π*_true_ + 0.1	*π*_true_ - 0.1 *π*_true_ + 0.2 *π*_true_ + 0.3
True discordant results diseased population ${\psi}_{{\mathrm{D}}_{\mathrm{true}}}$	0.11 (0.15, if: Se_E_ − Se_C_ = 0.15)	0.18, 0.26
Assumed discordant results diseased population ${\psi}_{{\mathrm{D}}_{\mathrm{ass.}}}$	0.18	
True discordant results non-diseased population ${\psi}_{{\mathrm{ND}}_{\mathrm{true}}}$	0.14 (0.15, if: Sp_E_ − Sp_C_ = 0.15)	0.24, 0.38
Assumed discordant results non-diseased population ${\psi}_{{\mathrm{ND}}_{\mathrm{ass.}}}$	0.24	
Sensitivity experimental test Se_E_	$\hat{=}\mathrm{S}{\mathrm{e}}_{\mathrm{C}}$	
Specificity experimental test Sp_E_	$\hat{=}\mathrm{S}{\mathrm{p}}_{\mathrm{C}}$	
Sensitivity experimental test Se_E_	Se_C_ + 0.1	Se_C_ + 0.05 Se_C_ + 0.15
Specificity experimental test Sp_E_	Sp_C_ + 0.1	Sp_C_ + 0.05 Sp_C_ + 0.15

Table 5 shows distributions involved in the data generation mechanism. We use the statistical software *R* version 4.0.5 to perform the simulations with the default random number generator Mersenne-Twister, but with the own initialization methods of *R* [28, 29].

**Table 5:** Description of the data generation mechanism of the unpaired and paired design in the simulation study (*Bin*: binomial distribution, *MVBin*: multivariate binomial distribution, *k*: number of trials, *p*: success probability, *ρ*: dependence between both tests, *N*: total sample size, *n*_*DE*_: diseased individuals in experimental group, *n*_*DC*_: diseased individuals in comparator group)

	Unpaired design	Paired design
Diseased individuals (n_D_) according to reference standard	${n}_{D_E}\;\sim\;Bin(k = N, p = \pi_\mathrm{true})$ $n_{D_C}\,\sim\,Bin(k = N, p = \pi_\mathrm{true})$	*n*_*D *_~ *Bin*(*k* = *N*, *p* = *π*_true_)
True Positive Results (TP)	$TP_{E}\,\sim Bin(k = n_{D_E}, p = Se_{E})$ $TP_{C}\,\sim Bin(k = n_{D_C}, p = Se_{C})$	$(TP_{E}, TP_{C})\,\sim MVBin(k_\mathrm{E} = n_{D_E}, k_\mathrm{C} = n_{D_C},$ *p*_E_ = *Se*_*E*_, *p*_C_ = *Se*_*C*_, *ρ* = *TPPR*)
True Negative Results (TN)	$TN_{E}\,\sim Bin(k = N - n_{D_E}, p = Sp_{E})$ $TN_{C}\,\sim Bin(k = N - n_{D_C}, p = Sp_{C})$	$(TN_{E}, TN_{C})\,\sim MVBin(k_\mathrm{E} = N - n_{D_E}, k_\mathrm{C} = N - n_{D_C},$ *p*_E_ = *Sp*_*E*_, *p*_C_ = *Sp*_*C*_, *ρ* = *TNNR*)

**Fig. 4:**
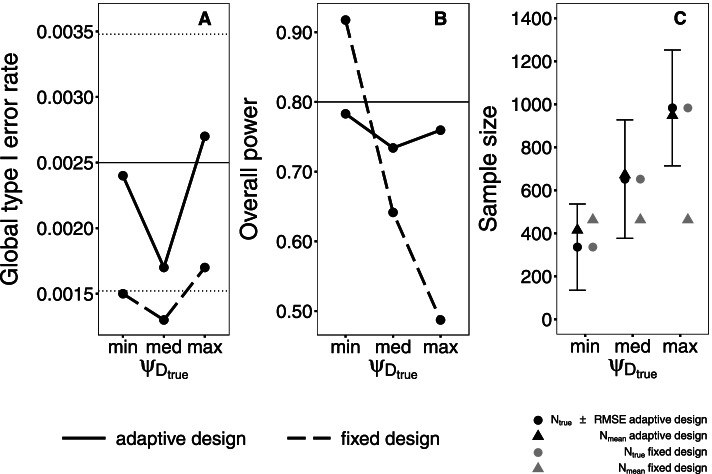
Global type I error, overall power and sample sizes of the fixed and adaptive paired design. Simulations are based on the initial scenario and a variation of the true proportion of discordant test results in the diseased population (${\psi}_{{\mathrm{D}}_{\mathrm{true}}}$). In Fig. A, black dotted lines mark the interval of Monte Carlo error due to simulations. In Fig. B, the target power equals 0.8

Figure 4 shows type I error rates with according Monte Carlo errors due to simulations (1.96 x SE = 0.00098), power and true sample sizes (*N*_true_) with root-mean-squared-error of the re-estimated sample size (RMSE) under H_1_ and additionally the mean of the re-estimated samples sizes per scenario (*N*_mean_) of those scenarios containing the minimal, medium and maximal ${\psi}_{{\mathrm{D}}_{\mathrm{true}}}$ in the paired study design**.** The depicted results offer some characteristics which can be generalized to other scenarios in the paired and unpaired design. Referring to Fig. A, one important aspect is that scenarios preserve type I error rates. In analogy to the overall power of the Intersection-Union Test explained in section 2, global type I error rates result as the product of the individual type I error rates of each endpoint (0.05 two-sided each). Due to the analysis with the score confidence interval in this scenario with small prevalence, results are conservative [24].

Considering Fig. B and C, the overall power of the fixed design decreases with increasing ${\psi}_{{\mathrm{D}}_{\mathrm{true}}}$. The larger ${\psi}_{{\mathrm{D}}_{\mathrm{true}}}$ is, the smaller the dependence between both tests is. The smaller the dependence between both tests is, the larger *N*_true_ becomes. The discrepancy between *N*_true_ and *N*_mean_ in the fixed design increases, if ${\psi}_{{\mathrm{D}}_{\mathrm{true}}}$ increases. If ${\psi}_{{\mathrm{D}}_{\mathrm{true}}}$ is medium, the assumption about this parameter in the fixed design equals the true parameter. But the assumption about the prevalence is larger than the prevalence is in truth. Therefore, *N*_mean_ is smaller than *N*_true_ and the overall power is smaller than the target power of 80%.

The adaptive design compensates wrong assumptions about nuisance parameters. The discrepancy between *N*_true_ and *N*_mean_ of the adaptive design is small. Hence, the overall power comes close to the target power. The adaptive design re-estimates ${\psi}_{{\mathrm{D}}_{\mathrm{true}}}$, ${\psi}_{{\mathrm{ND}}_{\mathrm{true}}}$ and *π*_true_ without any relevant bias. In those scenarios based on the initial prevalence of 20%, relative bias of ${\hat{\psi}}_{\mathrm{D}}$ is little higher than relative bias of ${\hat{\psi}}_{\mathrm{ND}}$. Due to this prevalence, there is only a small number of diseased patients in the sample which can be consulted for the re-estimation of ${\psi}_{{\mathrm{D}}_{\mathrm{true}}}$. Supplement materials show simulations results of the bias.

**Fig. 5:**
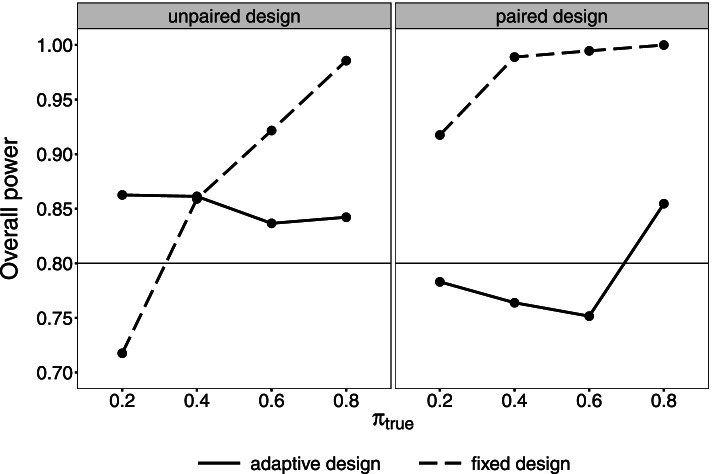
Overall power of the fixed and adaptive design in an unpaired and paired diagnostic study. Simulations are based on the initial scenario and a variation of the true prevalence (*π*_true_). The target power equals 0.8

Figure 5 compares the overall power depending on the true prevalence *π*_true_ in the unpaired and paired design. If *π*_true_ is low, the power in both fixed designs is the lowest. The power becomes larger with increasing prevalence. In the depicted scenarios, the assumed prevalence is larger than the true prevalence. A low true prevalence represents a small number of diseased individuals. In this case, the number of diseased individuals is the determining aspect for sample size calculation to show the sensitivity. In the fixed unpaired design, a higher number of diseased individuals is wrongly assumed which results in a too small sample size and power. Vice versa, a high true prevalence leads to a too large sample size and power. The number of non-diseased individuals now determines the sample size to show the specificity. Due to the wrongly assumed prevalence, a too small number of non-diseased individuals is expected. The sample size is calculated too large. The fixed paired design is highly overpowered, independent of *π*_true_. Both proportions of discordant test results are assumed higher than in truth. The sample size is calculated too large.

In contrast to the fixed designs, both adaptive designs reveal a power closer to the target power of 80%. If *π*_true_ equals 80%, the overall power of the adaptive paired design stands out. In this scenario, the proportion of non-diseased individuals is initially assumed smaller than in truth. Hence, the sample size used for the re-estimation of nuisance parameters is already larger than the true sample size. The overall power is higher compared to scenarios with a lower *π*_true_.

## Discussion

In this article, we present an approach for blinded sample size re-estimation in a comparative diagnostic accuracy study. This allows the sample size to be revised for incorrect assumptions during the course of the study, so that the study is neither over- nor underpowered. We use an example and simulation study to show that the approach does not inflate type I error rates, reach the target power and re-estimate nuisance parameters without any relevant bias.

One strength of our simulation study is that it is based on a realistic initial scenario. Therefore, the simulation study covers the results of realistic as well as of extreme parameter combinations. But of course the simulation study does not depict all possible parameter combinations.

One general weakness of our proposed approach is that the sample size calculation and the confidence intervals used for evaluation are not based on the same formulas.

McCray et al. [11] use a sample size calculation and an evaluation method which belong together. Due to different endpoints in the approach of McCray et al. [11] and our approach, we don’t compare both approaches within an extensive simulation study. However, we compare both approaches within the example study. We show that our approach requires a smaller sample size and comes closer to the target power than the approach of McCray et al. [11], if the dependence between both diagnostic tests is maximal. In contrast to our work, McCray et al. [11] do not extend their approach to show non-inferiority or a combination of superiority and non-inferiority in both diagnostic tests.

We recommend to apply blinded adaptive designs in comparative diagnostic accuracy studies, especially if the nuisance parameters are extremely small or large. The reason for this is that a blinded adaptive design can correct extremely small or large sample sizes based on wrong assumptions.

Our work creates some space for further research. One important unanswered question asks about the consequences of the re-estimation of the prevalence on the blinding if predictive values are chosen as co-primary endpoints. Both, the positive and negative predictive value depend on the prevalence. Hence, the analysis is not blinded in the strong sense. Furthermore, it is of interest to develop unblinded adaptive designs in comparative diagnostic accuracy studies to allow for early stopping due to futility or efficacy [9].

## Conclusions

A confirmatory diagnostic accuracy study can either be performed as a single-test or a comparative study design. Comparative study designs are distinguished between an unpaired and paired study design. Stark et al. [8] introduce the optimal sample size calculation and the blinded adaptive design to re-estimate the sample size in the single-test design. This approach avoids an overpowered diagnostic accuracy study by calculating the sample size for two co-primary endpoints sensitivity and specificity in dependence of the prevalence of the disease.

In this article, we transfer the optimal sample size calculation to both comparative study designs. Furthermore, we propose blinded adaptive designs for an unpaired and paired diagnostic accuracy study. In the unpaired design, the adaptive design re-estimates the prevalence whereas, in the paired design, it additionally re-estimates the proportions of discordant test results. Subsequent to the re-estimation of these nuisance parameters, the sample size is re-calculated. Due to the blinded character of the adaptive designs, type I error rates are not inflated. Both approaches reach the target power and re-estimate nuisance parameters without any relevant bias.

We recommend to apply the optimal sample size calculation and a blinded adaptive design in a confirmatory diagnostic accuracy trial. Both approaches support to calculate the necessary sample size to achieve the targeted power without much additional effort.

## Supplementary Information

**Additional file 1.** Formulas for the optimal sample size calculation.

**Additional file 2.** R-Code for the optimal sample size calculation testing for superiority in both endpoints in the unpaired and paired design.

**Additional file 3 **Figure containing the comparison of the optimal sample size calcul**a**tion with the approach of McCray et al. [11].

**Additional file 4.** Simulation results of the blinded sample size re-estimation in the unpaired design.

**Additional file 5.** Simulation results of the blinded sample size re-estimation in the paired design.

## Data Availability

All simulations results used to illustrate the method can be found in online additional material of this article. This additional material is available online for the article: - Additional file 1 (“Additional_file_1_pdf): Formulas for the optimal sample size calculation - Additional file 2 (“Additional_file_2.pdf”): R-Code for the optimal sample size calculation testing for superiority in both endpoints in the unpaired and paired design - Additional file 3 (“Additional_file_3.pdf”): Figure containing the comparison of the optimal sample size calculation with the approach of McCray et al. [11] - Additional file 4 (“Additional_file_4.pdf”): Simulation results of the blinded sample size re-estimation in the unpaired design - Additional file 5 (“Additional_file_5.pdf”): Simulation results of the blinded sample size re-estimation in the paired design

## Abbreviations

Bin: Binomial distribution
CT: Computed Tomography
MVBin: Multivariate Binomial distribution
PET: Positron-Emission Tomography
RMSE: Root-Mean-Squared-Error
Se_C_: Sensitivity of the comparator test
Se_E_: Sensitivity of the experimental test
Sp_C_: Specificity of the comparator test
Sp_E_: Specificity of the experimental test
TNNR: True-Negative-Negative-Rate
TPPR: True-Positive-Positive-Rate

## References

[1] Zhou X-H, McClish DK, Obuchowski NA (2011). Statistical methods in diagnostic medicine.

[2] Pepe MS (2003). The statistical evaluation of medical tests for classification and prediction.

[3] Bossuyt PM, Irwig L, Craig J, Glasziou P (2006). Comparative accuracy: assessing new tests against existing diagnostic pathways. BMJ.

[4] Committee for Medicinal Products for Human Use (CHMP). Guideline on clinical evaluation of diagnostic agents. London: European Medicines Agency, https://www.ema.europa.eu/en/documents/scientific-guideline/guideline-clinical-evaluation-diagnostic-agents_en.pdf. Accessed 21 March 2021.

[5] U.S. Food and Drug Administration (FDA). Guidance for industry and FDA staff: statistical guidance on reporting results from studies evaluating diagnostic tests. https://www.fda.gov/regulatory-information/search-fda-guidance-documents/statistical-guidance-reporting-results-studies-evaluating-diagnostic-tests-guidance-industry-and-fda. Accessed 21 March 2021.

[6] Hamasaki T, Evans SR, Asakura K (2018). Design, data monitoring, and analysis of clinical trials with co-primary endpoints: a review. J Biopharm Stat.

[7] Korevaar DA, Gopalakrishna G, Cohen JF, Bossuyt PM (2019). Targeted test evaluation: a framework for designing diagnostic accuracy studies with clear study hypotheses. Diagn Prognostic Res.

[8] Stark M, Zapf A (2020). Sample size calculation and re-estimation based on the prevalence in a single-arm confirmatory diagnostic accuracy study. Stat Methods Med Res.

[9] Zapf A, Stark M, Gerke O, Ehret C, Benda N, Bossuyt P, Deeks J, Reitsma J, Alonzo T, Friede T (2020). Adaptive trial designs in diagnostic accuracy research. Stat Med.

[10] Mazumdar M, Liu A (2003). Group sequential design for comparative diagnostic accuracy studies. Stat Med.

[11] McCray GP, Titman AC, Ghaneh P, Lancaster GA (2017). Sample size re-estimation in paired comparative diagnostic accuracy studies with a binary response. BMC Med Res Methodol.

[12] Thomopoulos NT (2017). Statistical distributions.

[13] Hajian-Tilaki K (2014). Sample size estimation in diagnostic test studies of biomedical informatics. J Biopharm Inform.

[14] Flahault A, Cadilhac M, Thomas G (2005). Sample size calculation should be performed for design accuracy in diagnostic test studies. J Clin Epidemiol.

[15] Buderer NM (1996). Statistical methodology: I. incorporating the prevalence of disease into the sample size calculation for sensitivity and specificity. Acad Emerg Med.

[16] Miettinen OS (1968). The matched pairs design in the case of all-or-none responses. Biometrics.

[17] Miettinen O, Nurminen M (1985). Comparative analysis of two rates. Stat Med.

[18] Agresti A (2013). Categorical data analysis.

[19] Agresti A, Caffo B (2000). Simple and effective confidence intervals for proportions and differences of proportions result from adding two successes and two failures. Am Stat.

[20] Agresti A, Coull BA (1998). Approximate is better than “exact” for interval estimation of binomial proportions. Am Stat.

[21] Connor RJ (1987). Sample size for testing differences in proportions for the paired-sample design. Biometrics.

[22] Agresti A, Min Y (2005). Simple improved confidence intervals for comparing matched proportions. Stat Med.

[23] Tango T (1998). Equivalence test and confidence interval for the difference in proportions for the paired-sample design. Stat Med.

[24] Fagerland MW, Lydersen S, Laake P (2014). Recommended tests and confidence intervals for paired binomial proportions. Stat Med.

[25] Brown LD, Cai TT, DasGupta A (2001). Interval estimation for a binomial proportion. Stat Sci.

[26] Held L, Sabanés BD (2014). Applied statistical inference.

[27] Alonzo TA, Pepe MS, Moskowitz CS (2002). Sample size calculations for comparative studies of medical tests for detecting presence of disease. Stat Med.

[28] Matsumoto M, Nishimura T (1998). Mersenne twister: a 623-dimensionally equidistributed uniform pseudo-random number generator. ACM Trans Model Comput Simulation (TOMACS).

[29] R Core Team: R (2021). A language and environment for statistical computing. In.

