# Supplementary material for: Blinded sample size re-estimation in a comparative diagnostic accuracy study
Source: BMC Med Res Methodol. 2022 Apr 19;22:115. doi: 10.1186/s12874-022-01564-2 (PMC9019976; doi:10.1186/s12874-022-01564-2)
Supplement: Supplementary file 1 — Additional file 1. Formulas for the optimal sample size calculation. [file 12874_2022_1564_MOESM1_ESM.pdf]

Appendix A Optimal sample size calculation

A.I. Optimal sample size calculation to show superiority in sensitivity and non-inferiority in specificity

To investigate if the experimental test is superior to the comparator test regarding sensitivity and non-inferior regarding specificity, $\mathrm{H}_{0_{\mathrm{global}}}$ is defined as:

$$\mathrm{H}_{0_{\mathrm{global}}}\colon\ \mathrm{H}_{0_{\mathrm{Se}}}\colon \mathrm{Se_E} = \mathrm{Se_C}\ \cup\ \mathrm{H}_{0_{\mathrm{Sp}}}\colon \mathrm{Sp_E} \leq \mathrm{Sp_C} - \Delta$$

The positive non-inferiority margin is denoted by Δ.

A.I.I. Unpaired design

To calculate the corresponding sample size in the unpaired design, the formulas for superiority and non-inferiority following Zhou et al. [1] are combined:

$$\frac{\left(z_{\alpha/2}\sqrt{V_0(\mathrm{Se_C}-\mathrm{Se_E})} + z_{\beta_{\mathrm{Se}}}\sqrt{V_A(\mathrm{Se_C}-\mathrm{Se_E})}\right)^2}{(\mathrm{Se_C}-\mathrm{Se_E})^2 \cdot \pi} \stackrel{!}{=} \frac{\left(\mathrm{z}_{\alpha/2} + z_{\beta_{\frac{1-\beta_{\mathrm{Se}}-\mathrm{Power_{overall}}}{1-\beta_{\mathrm{Se}}}}}\right)^2 \cdot V_A(\mathrm{Sp_C}-\mathrm{Sp_E})}{(\mathrm{Sp_C}-\mathrm{Sp_E}-\Delta)^2 \cdot (1-\pi)}$$

In the following formulas, α denotes the two-sided type I error rate.

A.I.II. Paired design

In the paired design, the optimal sample size calculation combines the formula for superiority of Miettinen et al. [2] and the formula for non-inferiority of Liu et al. [3]. Liu et

al. [3] report the sample size formula to test for equivalence. In appendix B, the sample size formula to test for non-inferiority based on the power function of Liu et al. [3] is derived.

$$\frac{\left(z_{1-\alpha/2}\cdot\psi_{\mathrm{D}}+z_{1-\beta_{\mathrm{Se}}}\sqrt{\psi_{\mathrm{D}}^2-\frac{1}{4}(\mathrm{Se_C}-\mathrm{Se_E})^2(3+\psi_{\mathrm{D}})}\right)^2}{\psi_D(\mathrm{Se_C}-\mathrm{Se_E})^2\pi}\overset{!}{=}$$

$$\frac{(\psi_{\mathrm{ND}}-(\mathrm{Sp_E}-\mathrm{Sp_C})^2)\left(\frac{z_{\alpha/2}}{w_{u_{\mathrm{Sp}}}}+z_{\frac{1-\beta_{\mathrm{Se}}-\mathrm{Power_{overall}}}{1-\beta_{\mathrm{Se}}}}\right)^2}{\left(-\Delta-(\mathrm{Sp_E}-\mathrm{Sp_C})\right)^2\cdot(1-\pi)}$$

With

$$w_{u_{\mathrm{Sp}}}=\frac{\sqrt{2p_{01}+(\mathrm{Sp_E}-\mathrm{Sp_C})-(\mathrm{Sp_E}-\mathrm{Sp_C})^2}}{\sqrt{2\cdot\overline{p}_{u,01}-\Delta-\Delta^2}}$$

$$\overline{p}_{u,01}=\frac{\left(-a_u+\sqrt{a_u^2-8b_u}\right)}{4}$$

$$a_u=\ -(\mathrm{Sp_E}-\mathrm{Sp_C})(1-\Delta)-2(p_{01}+\Delta)$$

$$b_u=\Delta(1+\Delta)p_{01}$$

$$p_{01}=\frac{\psi-\mathrm{Sp_E}+\mathrm{Sp_C}}{2}$$

A.II. Optimal sample size calculation to show non-inferiority in sensitivity and superiority in specificity

To investigate if the experimental test is non-inferior to the comparator test regarding sensitivity and superior regarding specificity, $\mathrm{H}_{0_{\mathrm{global}}}$ is defined as:

$$\mathrm{H}_{0_{\mathrm{global}}}:\ \mathrm{H}_{0_{\mathrm{Se}}}:\mathrm{Se_E}\le\mathrm{Se_C}-\Delta\cup\ \mathrm{H}_{0_{\mathrm{Sp}}}:\mathrm{Sp_E}=\mathrm{Sp_C}$$

The positive non-inferiority margin is denoted by Δ.

A.II.I. Unpaired design

$$\frac{\left(z_{\alpha/2}+z_{\beta_{\mathrm{Se}}}\right)^2\cdot V_A(\mathrm{Se_C}-\mathrm{Se_E})}{(\mathrm{Se_C}-\mathrm{Se_E}-\Delta)^2\cdot\pi}\overset{!}{=}$$

$$\frac{\left(z_{\alpha/2}\sqrt{V_0(\mathrm{Sp_C}-\mathrm{Sp_E})}+z_{\frac{1-\beta_{\mathrm{Se}}-\mathrm{Power_{overall}}}{1-\beta_{\mathrm{Se}}}}\sqrt{V_A(\mathrm{Sp_C}-\mathrm{Sp_E})}\right)^2}{(\mathrm{Sp_C}-\mathrm{Sp_E})^2\cdot(1-\pi)}$$

A.II.II. Paired design

$$\frac{(\psi_{\mathrm{D}}-(\mathrm{Se_E}-\mathrm{Se_C})^2)\left(\frac{z_{\alpha/2}}{w_{u_{\mathrm{Se}}}}+z_{\beta_{\mathrm{Se}}}\right)^2}{\left(-\Delta-(\mathrm{Se_E}-\mathrm{Se_C})\right)^2\cdot\pi}\overset{!}{=}$$

$$\frac{\left(z_{1-\alpha/2}\cdot\psi_{\mathrm{ND}}+z_{1-\frac{1-\beta_{\mathrm{se}}-\mathrm{Power_{overall}}}{1-\beta_{\mathrm{se}}}}\sqrt{\psi_{\mathrm{ND}}^2-\frac{1}{4}(\mathrm{Sp_C}-\mathrm{Sp_E})^2(3+\psi_{\mathrm{ND}})}\right)^2}{\psi_{\mathrm{ND}}(\mathrm{Sp_C}-\mathrm{Sp_E})^2(1-\pi)}$$

The parameter $w_{u_{\mathrm{Se}}}$ is defined in analogy to $w_{u_{\mathrm{Sp}}}$above.

A.III. Optimal sample size calculation to show non-inferiority in both endpoints

To investigate if the experimental test is non-inferior to the comparator test regarding sensitivity and specificity, $\mathrm{H}_{0_{\mathrm{global}}}$ is defined as:

$$\mathrm{H}_{0_{\mathrm{global}}}:\ \mathrm{H}_{0_{\mathrm{Se}}}:\mathrm{Se_E}\leq\mathrm{Se_C}-\Delta_{\mathrm{Se}}\cup\ \mathrm{H}_{0_{\mathrm{Sp}}}:\mathrm{Sp_E}\leq\mathrm{Sp_C}-\Delta_{\mathrm{Sp}}$$

The positive non-inferiority margins are denoted by Δ_{Se} and Δ_{Sp}.

A.III.I. Unpaired design

$$\frac{\left(z_{\alpha/2}+z_{\beta_{\mathrm{Se}}}\right)^2\cdot V_A(\mathrm{Se_C}-\mathrm{Se_E})}{(\mathrm{Se_C}-\mathrm{Se_E}-\Delta)^2\cdot\pi}\overset{!}{=}\frac{\left(z_{\alpha/2}+z_{\beta_{\frac{1-\beta_{\mathrm{Se}}-\mathrm{Power_{overall}}}{1-\beta_{\mathrm{Se}}}}}\right)^2\cdot V_A(\mathrm{Sp_C}-\mathrm{Sp_E})}{(\mathrm{Sp_C}-\mathrm{Sp_E}-\Delta)^2\cdot\pi}$$

A.III.II. Paired design

$$\frac{(\psi_{\mathrm{D}} - (\mathrm{Se_E} - \mathrm{Se_C})^2)\left(\frac{z_{\alpha/2}}{w_{u_{\mathrm{Se}}}} + z_{\beta_{\mathrm{Se}}}\right)^2}{\left(-\Delta_{\mathrm{Se}} - (\mathrm{Se_E} - \mathrm{Se_C})\right)^2 \cdot \pi} \stackrel{!}{=} \frac{(\psi_{\mathrm{ND}} - (\mathrm{Sp_E} - \mathrm{Sp_C})^2)\left(\frac{z_{\alpha/2}}{w_{u_{\mathrm{Sp}}}} + z_{\frac{1-\beta_{\mathrm{se}}-\mathrm{Power_{overall}}}{1-\beta_{\mathrm{se}}}}\right)^2}{\left(-\Delta_{\mathrm{Sp}} - (\mathrm{Sp_E} - \mathrm{Sp_C})\right)^2 \cdot (1-\pi)}$$

Appendix B Derivation of the sample size for testing for non-inferiority in the paired design

Liu et al. [3] report the asymptotic power function to test for non-inferiority between the sensitivity or specificity of experimental test (θ_{E}) and comparator test (θ_{C}). The asymptotic power function is solved for the sample size.

$$1 - \phi\left(-\frac{z_{\alpha/2}}{w_u} - \frac{\Delta + (\theta_{\mathrm{E}} - \theta_{\mathrm{C}})}{\sigma}\right) = 1 - \beta = \mathrm{Power}$$

$$\phi\left(-\frac{z_{\alpha/2}}{w_u} - \frac{\Delta + (\theta_{\mathrm{E}} - \theta_{\mathrm{C}})}{\sqrt{\frac{\psi - (\theta_{\mathrm{E}} - \theta_{\mathrm{C}})^2}{n}}}\right) = \beta$$

$$-\frac{z_{\alpha/2}}{w_u} - \frac{\Delta + (\theta_{\mathrm{E}} - \theta_{\mathrm{C}})}{\sqrt{\frac{\psi - (\theta_{\mathrm{E}} - \theta_{\mathrm{C}})^2}{n}}} = z_\beta$$

$$\frac{-\Delta - (\theta_{\mathrm{E}} - \theta_{\mathrm{C}})}{\sqrt{\frac{\psi - (\theta_{\mathrm{E}} - \theta_{\mathrm{C}})^2}{n}}} = z_\beta + \frac{z_{\alpha/2}}{w_u}$$

$$\sqrt{\frac{\psi - (\theta_{\mathrm{E}} - \theta_{\mathrm{C}})^2}{n}} = \frac{-\Delta - (\theta_{\mathrm{E}} - \theta_{\mathrm{C}})}{z_\beta + \frac{z_{\alpha/2}}{w_u}}$$

$$\sqrt{n} = \frac{\sqrt{\psi - (\theta_{\mathrm{E}} - \theta_{\mathrm{C}})^2}\left(z_\beta + \frac{z_{\alpha/2}}{w_u}\right)}{-\Delta - (\theta_{\mathrm{E}} - \theta_{\mathrm{C}})}$$

$$n = (\psi - (\theta_\mathrm{E} - \theta_\mathrm{C})^2)\left(\frac{\frac{z_{\alpha/2}}{w_u} + z_\beta}{-\Delta - (\theta_\mathrm{E} - \theta_\mathrm{C})}\right)^2$$

With

$$\Delta > 0$$
$$w_u = \frac{\sqrt{2p_{01} + (\theta_E - \theta_C) - (\theta_E - \theta_C)^2}}{\sqrt{2 \cdot \overline{p}_{u,01} - \Delta - \Delta^2}}$$
$$\overline{p}_{u,01} = \frac{\left(-a_u + \sqrt{a_u^2 - 8b_u}\right)}{4}$$
$$a_u = -(\theta_E - \theta_C)(1 - \Delta) - 2(p_{01} + \Delta)$$
$$b_u = \Delta(1 + \Delta)p_{01}$$
$$p_{01} = \frac{\psi - \theta_E + \theta_C}{2}$$

References

1. Zhou X-H, McClish DK, Obuchowski NA. Statistical methods in diagnostic medicine. Vol. 569. 2nd ed. Hoboken, New Jersey: John Wiley & Sons; 2011.
2. Miettinen OS. The matched pairs design in the case of all-or-none responses. Biometrics. 1968;24:339-352.
3. Liu Jp, Hsueh Hm, Hsieh E, Chen JJ. Tests for equivalence or non-inferiority for paired binary data. Stat Med. 2002;21:231-245.
