# Supplementary material for: Blinded sample size re-estimation in a comparative diagnostic accuracy study
Source: BMC Med Res Methodol. 2022 Apr 19;22:115. doi: 10.1186/s12874-022-01564-2 (PMC9019976; doi:10.1186/s12874-022-01564-2)
Supplement: Supplementary file 2 — Additional file 2. R-Code for the optimal sample size calculation testing for superiority in both endpoints in the unpaired and paired design. [file 12874_2022_1564_MOESM2_ESM.pdf]

R-Code

Optimal sample size calculation testing for superiority in sensitivity and specificity

1. Unpaired design

```
unpaired_superiority <- function (alpha, power, theta.se.c, theta.se.e, theta.sp.c, theta.sp.e, prev) {
  # parameter description
  # alpha: desired type I error rate per endpoint
  # power: desired overall power
  # theta.se.c: sensitivity of the comparator test
  # theta.se.e: sensitivity of the experimental test
  # theta.sp.c: specificity of the comparator test
  # theta.sp.e: specificity of the experimental test
  # prev:  prevalence

  # the variance function
  v <- function(theta.c, theta.e) {
    variance <- theta.c * (1 - theta.c) + theta.e * (1 - theta.e)
    return(variance)
  }

  # sample size calculation for one endpoint following Zhou et al. (2011)
  unpaired <- function(alpha, beta, theta.c, theta.e) {
    n <- ceiling((qnorm(alpha / 2) * sqrt(v(theta.c, theta.c)) + qnorm(beta) * sqrt(v(theta.c, theta.e))) ^
         2 / (theta.c - theta.e) ^ 2)
  }

  # calculate power for one endpoint
  calculate.power.unpaired <- function(n, alpha, theta.c, theta.e) {
    z <- (sqrt(n) * (theta.c - theta.e) - qnorm(alpha / 2) * sqrt(v(theta.c, theta.c))) / sqrt(v(theta.c,
         theta.e))
    power <- 1 - pnorm(z)
    return(power)
  }

  # function for the optimal sample size
  f <- function(alpha, power, beta.se, theta.se.c, theta.se.e, theta.sp.c, theta.sp.e, prev) {
    diff.n <- qnorm(beta.se) * sqrt(v(theta.se.c, theta.se.e)) * sqrt(1 - prev) * (theta.sp.c - theta.sp.e) -
              qnorm(1 - (power / (1 - beta.se))) * sqrt(v(theta.sp.c, theta.sp.e)) * sqrt(prev) * (theta.se.c
              - theta.se.e) - qnorm(alpha / 2) * sqrt(v(theta.sp.c, theta.sp.c)) * sqrt(prev) * (theta.se.c -
              theta.se.e) + qnorm(alpha / 2)*sqrt(v(theta.se.c, theta.se.c)) * sqrt(1 - prev) * (theta.sp.c -
              theta.sp.e)

    return(diff.n)
  }
```

```
  # approximate the sample size for beta.se and then calculate beta.sp
  beta.se <- uniroot(f, alpha = alpha, power = power, theta.se.c = theta.se.c, theta.se.e = theta.se.e,
                  theta.sp.c = theta.sp.c, theta.sp.e = theta.sp.e, prev = prev,
                   lower = 0, upper = 1-power)$root
  beta.sp <- (power+beta.se-1)/(beta.se-1)
  power.total <- (1-beta.se)*(1-beta.sp)

  # calculate the sample size with beta.se and beta.sp
  n.se <- unpaired(alpha = alpha, beta = beta.se, theta.c = theta.se.c, theta.e = theta.se.e)
  n.sp <- unpaired(alpha = alpha, beta = beta.sp, theta.c = theta.sp.c, theta.e = theta.sp.e)
  N.se <- n.se / prev
  N.sp <- n.sp / (1 - prev)
  N <- ceiling(max(N.se, N.sp)) # total sample size per group

  # calculate total power
  power.se <- calculate.power.unpaired(n = N * prev, alpha = alpha, theta.c = theta.se.c, theta.e =
                                            theta.se.e)
  power.sp <- calculate.power.unpaired(n = N * (1 - prev), alpha = alpha, theta.c = theta.sp.c, theta.e
                                            = theta.sp.e)
  power.total <- power.se * power.sp
  return(list(N = N, N.se = N.se, N.sp = N.sp, power.total = power.total, power.se = power.se,
          power.sp = power.sp))
}
```

2. Paired design

```
paired_superiority <- function(alpha, power, theta.se.c, theta.se.e, theta.sp.c, theta.sp.e,
                                          psi.d, psi.nd, prev) {

 # sample size calculation for one endpoint following Miettinen (1968)
sample.size.paired.one.endpoint <- function(alpha, beta, theta.c, theta.e, psi){
   delta <- abs(theta.c-theta.e)
   n <- (qnorm(1-alpha/2)*psi+qnorm(1-beta)*sqrt((psi^2)-0.25*(delta^2)*(3+psi)))^2 /
        (psi*(delta^2))
   return(n)
  }

  # calculate power for one endpoint
  calculate.power <- function(n, alpha, theta.c, theta.e, psi) {
   delta <- abs(theta.c-theta.e)
   z <- (sqrt(n*psi)*delta - qnorm(1-alpha/2)*psi)/sqrt((psi^2)-0.25*(delta^2)*(3+psi))
   power <- pnorm(z)
   return(power)
  }

  # function for the equal sample size for both endpoints
  f <- function(alpha, power, beta.1, theta.se.c, theta.se.e, theta.sp.c, theta.sp.e, psi.d, psi.nd, prev) {
   delta.se <- abs(theta.se.c-theta.se.e)
   delta.sp <- abs(theta.sp.c-theta.sp.e)
   diff.n <- qnorm(1-beta.1)*sqrt((psi.d^2)-0.25*(delta.se^2)*(3+psi.d))*sqrt(psi.nd*(1-
             prev))*delta.sp - qnorm(power/(1-beta.1))*sqrt((psi.nd^2)-
             0.25*(delta.sp^2)*(3+psi.nd))*sqrt(psi.d*prev)*delta.se -
             qnorm(1-alpha/2)*psi.nd*sqrt(psi.d*prev)*delta.se + qnorm(1-
             alpha/2)*psi.d*sqrt(psi.nd*(1-prev))*delta.sp
   return(diff.n)
  }

  # solve the sample size for beta.1 and then calculate beta.2
  beta.1 <- uniroot(f, alpha = alpha, power = power, theta.se.c = theta.se.c, theta.se.e = theta.se.e,
             theta.sp.c = theta.sp.c, theta.sp.e = theta.sp.e, psi.d = psi.d, psi.nd = psi.nd,
             prev = prev, lower = 0, upper = 1-power)$root
  beta.2 <- (power+beta.1-1)/(beta.1-1)
  power.total.theoretical <- (1-beta.1)*(1-beta.2)

  # calculate sample size with known beta.1 and beta.2
  n.se <- sample.size.paired.one.endpoint(alpha = alpha, beta = beta.1, theta.c = theta.se.c, theta.e =
          theta.se.e, psi = psi.d)
  n.sp <- sample.size.paired.one.endpoint(alpha = alpha, beta = beta.2, theta.c = theta.sp.c, theta.e =
          theta.sp.e, psi = psi.nd)
  N.se <- n.se/prev
  N.sp <- n.sp/(1-prev)
  N <- ceiling(max(N.se, N.sp))
```

```
# calculate power
power.se <- calculate.power(n= N*prev, alpha = alpha, theta.c = theta.se.c, theta.e = theta.se.e, psi =
 psi.d)
power.sp <- calculate.power(n= N*(1-prev), alpha = alpha, theta.c = theta.sp.c, theta.e = theta.sp.e,
psi = psi.nd)
power.total <- power.se * power.sp

return(list(N = N, N.se = N.se, N.sp = N.sp, power.total = power.total, power.se = power.se, power.sp
= power.sp, beta.1 = beta.1, beta.2 = beta.2))
}
```
