# Supplementary figures and images for: Blinded sample size re-estimation in a comparative diagnostic accuracy study
Source: BMC Med Res Methodol. 2022 Apr 19;22:115. doi: 10.1186/s12874-022-01564-2 (PMC9019976; doi:10.1186/s12874-022-01564-2)

Empirical power

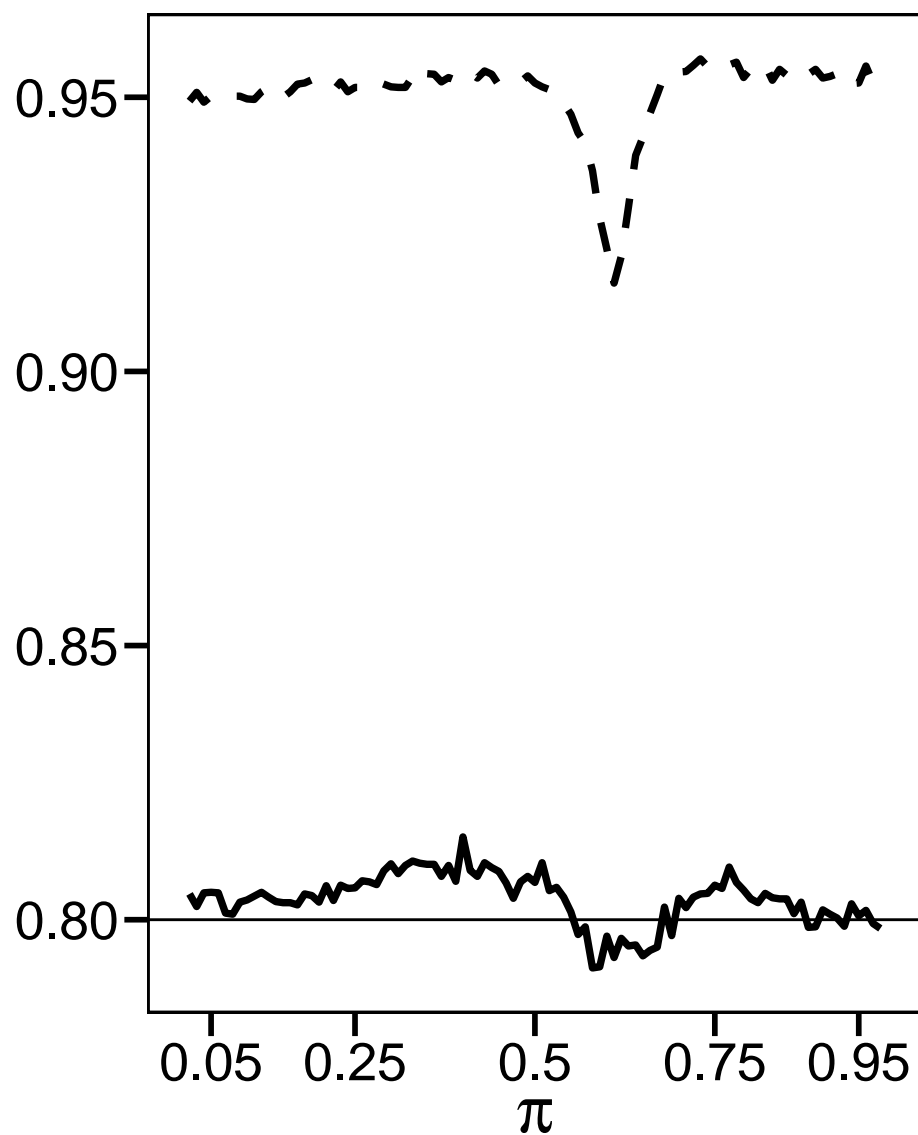

— optimal approach

Sample size

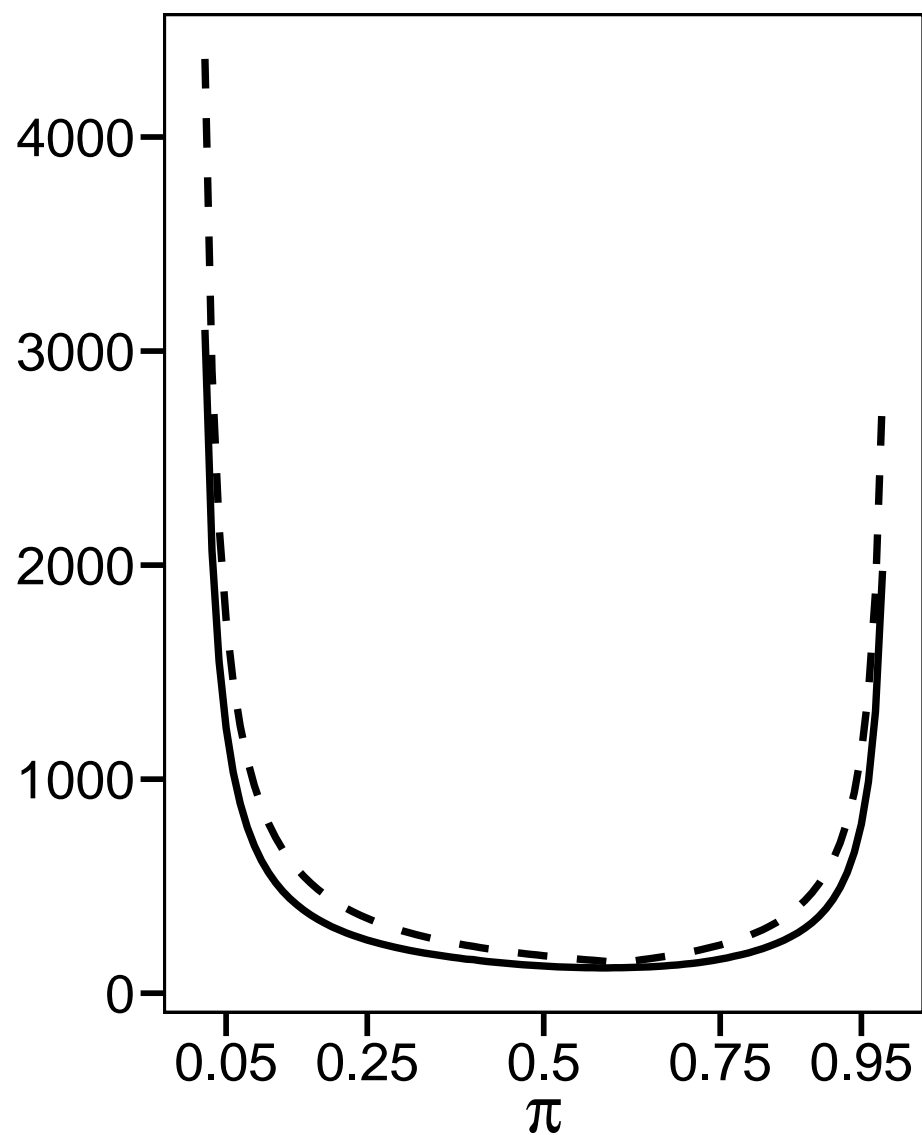

- - McCray et al.

Supplement: Supplementary file 3 — Additional file 3 Figure containing the comparison of the optimal sample size calculation with the approach of McCray et al. [11]. [file 12874_2022_1564_MOESM3_ESM.pdf]
