# Supplementary material for: Blinded sample size re-estimation in a comparative diagnostic accuracy study
Source: BMC Med Res Methodol. 2022 Apr 19;22:115. doi: 10.1186/s12874-022-01564-2 (PMC9019976; doi:10.1186/s12874-022-01564-2)
Supplement: Supplementary file 4 — Additional file 4. Simulation results of the blinded sample size re-estimation in the unpaired design. [file 12874_2022_1564_MOESM4_ESM.pdf]

Unpaired design

1. Testing for superiority in sensitivity and specificity (for hypothesis see manuscript, section 2)

Type I error rate

Se_C	Sp_C	Se_E	Sp_E	π_{true}	$\pi_{ass.}$	Type I error sensitivity fixed design	Type I error specificity fixed design	Type I error global fixed design	Type I error sensitivity adaptive design	Type I error specificity adaptive design	Type I error global adaptive design
0.81	0.66	0.9	0.8	0.44	0.47	0.0469	0.0445	0.002	0.0481	0.0468	0.0031
0.8	0.7	0.9	0.8	0.2	0.3	0.0527	0.0461	0.0016	0.0515	0.0486	0.0037
0.6	0.7	0.7	0.8	0.2	0.3	0.0478	0.0474	0.0021	0.0496	0.0482	0.0018
0.7	0.7	0.8	0.8	0.2	0.3	0.0529	0.0475	0.0025	0.0517	0.047	0.0023
0.8	0.6	0.9	0.7	0.2	0.3	0.0512	0.0514	0.0021	0.0511	0.0487	0.0026
0.8	0.8	0.9	0.9	0.2	0.3	0.054	0.0453	0.0031	0.0517	0.0476	0.002
0.8	0.7	0.9	0.8	0.4	0.5	0.0521	0.0427	0.0029	0.0513	0.0437	0.003
0.8	0.7	0.9	0.8	0.6	0.7	0.0487	0.0475	0.0029	0.0509	0.0478	0.0027
0.8	0.7	0.9	0.8	0.8	0.9	0.0479	0.0449	0.0021	0.051	0.0468	0.0027
0.8	0.7	0.9	0.8	0.2	0.1	0.0515	0.0462	0.0026	0.0489	0.0484	0.002
0.8	0.7	0.9	0.8	0.2	0.4	0.0522	0.0456	0.0019	0.0531	0.0451	0.002
0.8	0.7	0.9	0.8	0.2	0.5	0.0525	0.0447	0.002	0.053	0.0472	0.0028
0.8	0.7	0.85	0.8	0.2	0.3	0.0509	0.0508	0.0021	0.0495	0.0517	0.0035
0.8	0.7	0.95	0.8	0.2	0.3	0.0511	0.0446	0.0024	0.0519	0.0478	0.0028
0.8	0.7	0.9	0.75	0.2	0.3	0.0508	0.0462	0.003	0.0493	0.0455	0.0033
0.8	0.7	0.9	0.85	0.2	0.3	0.0533	0.0449	0.0021	0.0517	0.0469	0.0024

Power

Se_C	Sp_C	Se_E	Sp_E	π_{true}	$\pi_{ass.}$	Power sensitivity fixed design	Power specificity fixed design	Power overall fixed design	Power sensitivity adaptive design	Power specificitiy adaptive design	Power overall adaptive design
0.81	0.66	0.9	0.8	0.44	0.47	0.8604	0.988	0.8501	0.8782	0.9912	0.8706
0.8	0.7	0.9	0.8	0.2	0.3	0.7254	0.9891	0.7176	0.8636	0.9989	0.8626
0.6	0.7	0.7	0.8	0.2	0.3	0.646	0.9994	0.6455	0.8093	1	0.8093
0.7	0.7	0.8	0.8	0.2	0.3	0.6764	0.9984	0.6756	0.8335	1	0.8335
0.8	0.6	0.9	0.7	0.2	0.3	0.7492	0.9742	0.7301	0.869	0.9963	0.8656
0.8	0.8	0.9	0.9	0.2	0.3	0.7055	0.9992	0.7051	0.8663	1	0.8663
0.8	0.7	0.9	0.8	0.4	0.5	0.9235	0.9289	0.859	0.9251	0.9296	0.8613
0.8	0.7	0.9	0.8	0.6	0.7	0.9988	0.9228	0.9216	0.9927	0.8425	0.8366
0.8	0.7	0.9	0.8	0.8	0.9	1	0.9856	0.9856	1	0.8422	0.8422
0.8	0.7	0.9	0.8	0.2	0.1	0.9919	1	0.9919	0.8747	0.9992	0.8739
0.8	0.7	0.9	0.8	0.2	0.4	0.6675	0.9793	0.6544	0.8631	0.9989	0.862
0.8	0.7	0.9	0.8	0.2	0.5	0.6697	0.9787	0.6559	0.8662	0.9986	0.8648
0.8	0.7	0.85	0.8	0.2	0.3	0.6576	1	0.6576	0.8331	1	0.8331
0.8	0.7	0.95	0.8	0.2	0.3	0.9216	0.9159	0.845	0.9521	0.9451	0.9007
0.8	0.7	0.9	0.75	0.2	0.3	0.9742	0.8695	0.8467	0.9705	0.86	0.8348
0.8	0.7	0.9	0.85	0.2	0.3	0.7008	1	0.7008	0.8623	1	0.8623

Sample size and bias

Se_C	Sp_C	Se_E	Sp_E	π_{true}	$\pi_{ass.}$	true sample size	sample size fixed design	sample size interim analysis	sample size adaptive design	$\hat{\pi}$	bias = $\frac{\hat{\pi} - \pi_{true}}{\pi_{true}}$
0.81	0.66	0.9	0.8	0.44	0.47	653	624	312	656	0.4400	< 0.001
0.8	0.7	0.9	0.8	0.2	0.3	1175	830	415	1184	0.2000	-0.0002
0.6	0.7	0.7	0.8	0.2	0.3	1850	1240	620	1857	0.2000	0.0001
0.7	0.7	0.8	0.8	0.2	0.3	1590	1077	539	1602	0.2001	0.0003
0.8	0.6	0.9	0.7	0.2	0.3	1185	867	434	1191	0.2000	0.0002
0.8	0.8	0.9	0.9	0.2	0.3	1175	792	396	1184	0.2000	-0.0002
0.8	0.7	0.9	0.8	0.4	0.5	723	720	360	725	0.3997	-0.0009
0.8	0.7	0.9	0.8	0.6	0.7	818	1060	530	820	0.6001	0.0002
0.8	0.7	0.9	0.8	0.8	0.9	1591	3181	1591	1593	0.8000	< 0.0001
0.8	0.7	0.9	0.8	0.2	0.1	1175	2350	1175	1180	0.1999	-0.0003
0.8	0.7	0.9	0.8	0.2	0.4	1175	723	362	1185	0.2000	< 0.0001
0.8	0.7	0.9	0.8	0.2	0.5	1175	720	360	1185	0.2000	0.0001
0.8	0.7	0.85	0.8	0.2	0.3	4870	3247	1624	4876	0.2000	0.0002
0.8	0.7	0.95	0.8	0.2	0.3	580	510	255	584	0.2001	0.0004
0.8	0.7	0.9	0.75	0.2	0.3	1830	1877	939	1834	0.2000	-0.0001
0.8	0.7	0.9	0.85	0.2	0.3	1175	784	392	1183	0.1999	-0.0004

2. Testing for superiority in sensitivity and non-inferiority in specificity (for hypothesis see Additional file 1, section A.I)

Type I error rate

$\mathbf{Se_C}$	$\mathbf{Sp_C}$	$\mathbf{Se_E}$	$\mathbf{Sp_E}$	π_{true}	$\pi_{\text{ass.}}$	non-inferiority margin specificity	Type I error sensitivity fixed design	Type I error specificity fixed design	Type I error global fixed design	Type I error sensitivity adaptive design	Type I error specificity adaptive design	Type I error global adaptive design
0.8	0.7	0.9	0.7	0.2	0.3	0.1	0.0502	0.0229	0.0013	0.0503	0.022	0.0016
0.6	0.7	0.7	0.7	0.2	0.3	0.1	0.049	0.0248	0.0012	0.0522	0.027	0.001
0.7	0.7	0.8	0.7	0.2	0.3	0.1	0.0491	0.0241	0.0009	0.047	0.0236	0.0007
0.8	0.6	0.9	0.6	0.2	0.3	0.1	0.0501	0.0222	0.0008	0.05	0.0222	0.0014
0.8	0.8	0.9	0.8	0.2	0.3	0.1	0.0494	0.0253	0.0009	0.0486	0.0243	0.0011
0.8	0.7	0.9	0.7	0.4	0.5	0.1	0.052	0.0257	0.0011	0.0518	0.0261	0.0011
0.8	0.7	0.9	0.7	0.6	0.7	0.1	0.0501	0.0244	0.0014	0.0483	0.0263	0.0011
0.8	0.7	0.9	0.7	0.8	0.9	0.1	0.0519	0.0248	0.0016	0.0484	0.0259	0.0013
0.8	0.7	0.9	0.7	0.2	0.1	0.1	0.0527	0.0247	0.0009	0.0501	0.0232	0.0009
0.8	0.7	0.9	0.7	0.2	0.4	0.1	0.0513	0.0238	0.001	0.0492	0.0241	0.0013
0.8	0.7	0.9	0.7	0.2	0.5	0.1	0.0497	0.0243	0.0014	0.0523	0.0247	0.0011
0.8	0.7	0.85	0.7	0.2	0.3	0.1	0.0509	0.0252	0.0025	0.049	0.0243	0.0012
0.8	0.7	0.95	0.7	0.2	0.3	0.1	0.0501	0.0266	0.0014	0.0517	0.0244	0.0008
0.8	0.7	0.9	0.7	0.2	0.3	0.05	0.0562	0.025	0.0013	0.0562	0.0231	0.0012
0.8	0.7	0.9	0.7	0.2	0.3	0.15	0.0494	0.0238	0.0011	0.0503	0.0249	0.0014

Power

$\mathbf{Se_C}$	$\mathbf{Sp_C}$	$\mathbf{Se_E}$	$\mathbf{Sp_E}$	π_{true}	$\pi_{\text{ass.}}$	non-inferiority margin specificity	Power sensitivity fixed design	Power specificity fixed design	Power overall fixed design	Power sensitivity adaptive design	Power specificitiy adaptive design	Power overall adaptive design
0.8	0.7	0.9	0.7	0.2	0.3	0.1	0.7386	0.9805	0.7234	0.8751	0.9964	0.8722
0.6	0.7	0.7	0.7	0.2	0.3	0.1	0.6465	0.9979	0.6453	0.8165	1	0.8165
0.7	0.7	0.8	0.7	0.2	0.3	0.1	0.6747	0.9957	0.6721	0.8395	0.9999	0.8395
0.8	0.6	0.9	0.6	0.2	0.3	0.1	0.7582	0.9695	0.7337	0.8756	0.9925	0.8686
0.8	0.8	0.9	0.8	0.2	0.3	0.1	0.7197	0.9944	0.7152	0.8724	0.9988	0.8715
0.8	0.7	0.9	0.7	0.4	0.5	0.1	0.9287	0.9015	0.8382	0.931	0.9013	0.8401
0.8	0.7	0.9	0.7	0.6	0.7	0.1	0.9986	0.8951	0.8938	0.9946	0.8121	0.8076
0.8	0.7	0.9	0.7	0.8	0.9	0.1	1	0.9794	0.9794	1	0.8145	0.8145
0.8	0.7	0.9	0.7	0.2	0.1	0.1	0.9905	1	0.9905	0.8747	0.9975	0.8724
0.8	0.7	0.9	0.7	0.2	0.4	0.1	0.6866	0.966	0.6629	0.8722	0.9971	0.8697
0.8	0.7	0.9	0.7	0.2	0.5	0.1	0.6872	0.9655	0.6636	0.871	0.9963	0.8684
0.8	0.7	0.85	0.7	0.2	0.3	0.1	0.6589	1	0.6589	0.8289	1	0.8289
0.8	0.7	0.95	0.7	0.2	0.3	0.1	0.9321	0.8829	0.8213	0.9579	0.9198	0.8803
0.8	0.7	0.9	0.7	0.2	0.3	0.05	0.9722	0.8548	0.8304	0.9692	0.8478	0.8209
0.8	0.7	0.9	0.7	0.2	0.3	0.15	0.7114	1	0.7114	0.867	1	0.867

Sample size and bias

$\mathbf{Se_C}$	$\mathbf{Sp_C}$	$\mathbf{Se_E}$	$\mathbf{Sp_E}$	π_{true}	$\pi_{\text{ass.}}$	non-inferiority margin specificity	true sample size	sample size fixed design	sample size interim analysis	sample size adaptive design	$\hat{\pi}$	bias = $\frac{\hat{\pi} - \pi_{\text{true}}}{\pi_{\text{true}}}$
0.8	0.7	0.9	0.7	0.2	0.3	0.1	1180	844	422	1192	0.2	-0.0001
0.6	0.7	0.7	0.7	0.2	0.3	0.1	1850	1247	624	1867	0.1998	-0.0009
0.7	0.7	0.8	0.7	0.2	0.3	0.1	1615	1084	542	1610	0.2	-0.0001
0.8	0.6	0.9	0.6	0.2	0.3	0.1	1187	874	437	1200	0.2	-0.0001
0.8	0.8	0.9	0.8	0.2	0.3	0.1	1175	800	400	1191	0.2	-0.0002
0.8	0.7	0.9	0.7	0.4	0.5	0.1	739	742	371	743	0.3999	-0.0001
0.8	0.7	0.9	0.7	0.6	0.7	0.1	845	1100	550	849	0.5999	-0.0001
0.8	0.7	0.9	0.7	0.8	0.9	0.1	1651	3301	1651	1654	0.7998	-0.0002
0.8	0.7	0.9	0.7	0.2	0.1	0.1	1180	2350	1175	1184	0.2	< 0.0001
0.8	0.7	0.9	0.7	0.2	0.4	0.1	1180	739	370	1194	0.2001	0.0007
0.8	0.7	0.9	0.7	0.2	0.5	0.1	1180	742	371	1194	0.2001	0.0004
0.8	0.7	0.85	0.7	0.2	0.3	0.1	4870	3247	1624	4883	0.2	0.0001
0.8	0.7	0.95	0.7	0.2	0.3	0.1	590	524	262	600	0.2	< 0.0001
0.8	0.7	0.9	0.7	0.2	0.3	0.05	1855	1907	954	1859	0.1999	-0.0004
0.8	0.7	0.9	0.7	0.2	0.3	0.15	1175	784	392	1189	0.2	-0.0002

3. Testing for non-inferiority in sensitivity and superiority in specificity (for hypothesis see Additional file 1, section A.II)

Type I error rate

$\mathbf{Se_C}$	$\mathbf{Sp_C}$	$\mathbf{Se_E}$	$\mathbf{Sp_E}$	π_{true}	$\pi_{ass.}$	non-inferiority margin sensitivity	Type I error sensitivity fixed design	Type I error specificity fixed design	Type I error global fixed design	Type I error sensitivity adaptive design	Type I error specificity adaptive design	Type I error global adaptive design
0.8	0.7	0.8	0.8	0.2	0.3	0.1	0.0242	0.0484	0.0013	0.0241	0.0478	0.0014
0.6	0.7	0.6	0.8	0.2	0.3	0.1	0.0262	0.0496	0.0012	0.0251	0.0493	0.0011
0.7	0.7	0.7	0.8	0.2	0.3	0.1	0.0241	0.0476	0.0015	0.0238	0.0496	0.0013
0.8	0.6	0.8	0.7	0.2	0.3	0.1	0.0247	0.0499	0.0009	0.0235	0.0491	0.0012
0.8	0.8	0.8	0.9	0.2	0.3	0.1	0.0248	0.0521	0.0007	0.0252	0.0514	0.0005
0.8	0.7	0.8	0.8	0.4	0.5	0.1	0.0259	0.0495	0.0013	0.026	0.0486	0.0015
0.8	0.7	0.8	0.8	0.6	0.7	0.1	0.0256	0.0515	0.0009	0.0259	0.0524	0.0009
0.8	0.7	0.8	0.8	0.8	0.9	0.1	0.0255	0.0479	0.0015	0.0272	0.049	0.0011
0.8	0.7	0.8	0.8	0.2	0.1	0.1	0.0259	0.0498	0.0013	0.025	0.048	0.0008
0.8	0.7	0.8	0.8	0.2	0.4	0.1	0.0249	0.0482	0.0015	0.0246	0.0487	0.0008
0.8	0.7	0.8	0.8	0.2	0.5	0.1	0.0243	0.0484	0.0012	0.0274	0.0467	0.0014
0.8	0.7	0.8	0.8	0.2	0.3	0.05	0.0234	0.0463	0.0019	0.022	0.0469	0.0008
0.8	0.7	0.8	0.8	0.2	0.3	0.15	0.0274	0.0485	0.0016	0.0259	0.0495	0.0009
0.8	0.7	0.8	0.75	0.2	0.3	0.1	0.0288	0.0481	0.0014	0.0285	0.0485	0.0013
0.8	0.7	0.8	0.85	0.2	0.3	0.1	0.0235	0.0455	0.0007	0.0224	0.046	0.0006

Power

$\mathbf{Se_C}$	$\mathbf{Sp_C}$	$\mathbf{Se_E}$	$\mathbf{Sp_E}$	π_{true}	$\pi_{ass.}$	non-inferiority margin sensitivity	Power sensitivity fixed design	Power specificity fixed design	Power overall fixed design	Power sensitivity adaptive design	Power specificitiy adaptive design	Power overall adaptive design
0.8	0.7	0.8	0.8	0.2	0.3	0.1	0.6524	0.9928	0.647	0.8041	0.9995	0.8037
0.6	0.7	0.6	0.8	0.2	0.3	0.1	0.6356	0.9995	0.6353	0.8038	1	0.8038
0.7	0.7	0.7	0.8	0.2	0.3	0.1	0.6378	0.9981	0.6367	0.8059	1	0.8059
0.8	0.6	0.8	0.7	0.2	0.3	0.1	0.6654	0.9805	0.6509	0.8072	0.9957	0.8032
0.8	0.8	0.8	0.9	0.2	0.3	0.1	0.6384	0.9996	0.638	0.804	1	0.804
0.8	0.7	0.8	0.8	0.4	0.5	0.1	0.8591	0.9367	0.8048	0.8665	0.9389	0.8135
0.8	0.7	0.8	0.8	0.6	0.7	0.1	0.993	0.9205	0.9142	0.9773	0.8502	0.831
0.8	0.7	0.8	0.8	0.8	0.9	0.1	1	0.9846	0.9846	1	0.8443	0.8443
0.8	0.7	0.8	0.8	0.2	0.1	0.1	0.9761	1	0.9761	0.8052	0.9998	0.8051
0.8	0.7	0.8	0.8	0.2	0.4	0.1	0.5861	0.9796	0.5734	0.8091	0.9989	0.8085
0.8	0.7	0.8	0.8	0.2	0.5	0.1	0.5828	0.9797	0.5708	0.8095	0.9992	0.809
0.8	0.7	0.8	0.8	0.2	0.3	0.05	0.6283	1	0.6283	0.7988	1	0.7988
0.8	0.7	0.8	0.8	0.2	0.3	0.15	0.7923	0.9298	0.7365	0.8594	0.9584	0.8226
0.8	0.7	0.8	0.75	0.2	0.3	0.1	0.9258	0.8716	0.808	0.9266	0.8731	0.8095
0.8	0.7	0.8	0.85	0.2	0.3	0.1	0.6333	1	0.6333	0.8063	1	0.8063

Sample size and bias

$\mathbf{Se_C}$	$\mathbf{Sp_C}$	$\mathbf{Se_E}$	$\mathbf{Sp_E}$	π_{true}	$\pi_{ass.}$	non-inferiority margin sensitivity	true sample size	sample size fixed design	sample size interim analysis	sample size adaptive design	$\hat{\pi}$	bias = $\frac{\hat{\pi} - \pi_{true}}{\pi_{true}}$
0.8	0.7	0.8	0.8	0.2	0.3	0.1	1267	880	440	1277	0.2	-0.0001
0.6	0.7	0.6	0.8	0.2	0.3	0.1	1885	1264	632	1898	0.2	-0.0001
0.7	0.7	0.7	0.8	0.2	0.3	0.1	1650	1115	558	1666	0.2	< 0.0001
0.8	0.6	0.8	0.7	0.2	0.3	0.1	1265	917	459	1282	0.1999	-0.0006
0.8	0.8	0.8	0.9	0.2	0.3	0.1	1260	847	424	1274	0.1999	-0.0004
0.8	0.7	0.8	0.8	0.4	0.5	0.1	759	748	374	763	0.4	-0.0001
0.8	0.7	0.8	0.8	0.6	0.7	0.1	834	1064	532	838	0.6001	0.0002
0.8	0.7	0.8	0.8	0.8	0.9	0.1	1591	3181	1591	1594	0.7998	-0.0002
0.8	0.7	0.8	0.8	0.2	0.1	0.1	1267	2520	1260	1267	0.2001	0.0003
0.8	0.7	0.8	0.8	0.2	0.4	0.1	1267	759	380	1279	0.1999	-0.0007
0.8	0.7	0.8	0.8	0.2	0.5	0.1	1267	748	374	1279	0.1999	-0.0003
0.8	0.7	0.8	0.8	0.2	0.3	0.05	5025	3350	1675	5038	0.2	0.0001
0.8	0.7	0.8	0.8	0.2	0.3	0.15	635	544	272	643	0.2	-0.0001
0.8	0.7	0.8	0.75	0.2	0.3	0.1	1900	1901	951	1907	0.2	-0.0001
0.8	0.7	0.8	0.85	0.2	0.3	0.1	1260	840	420	1272	0.2001	0.0006

4. Testing for non-inferiority in sensitivity and specificity (for hypothesis see Additional file 1, section A.III)

Type I error rate

Se_C	Sp_C	Se_E	Sp_E	π_{true}	$\pi_{ass.}$	non-inferiority margin sensitivity	non-inferiority margin specificity	Type I error sensitivity fixed design	Type I error specificity fixed design	Type I error global fixed design	Type I error sensitivity adaptive design	Type I error specificity adaptive design	Type I error global adaptive design
0.2	0.7	0.8	0.7	0.2	0.3	0.1	0.1	0.0138	0.0127	0.0002	0.0137	0.0136	0.0002
0.6	0.7	0.6	0.7	0.2	0.3	0.1	0.1	0.0123	0.0119	0.0001	0.0124	0.0133	0.0002
0.8	0.7	0.8	0.7	0.2	0.3	0.1	0.1	0.0127	0.0127	0	0.0142	0.0135	0.0002
0.8	0.6	0.8	0.6	0.2	0.3	0.1	0.1	0.0127	0.014	0.0004	0.014	0.0133	0
0.8	0.8	0.8	0.8	0.2	0.3	0.1	0.1	0.0129	0.0118	0.0002	0.0125	0.0121	0.0003
0.8	0.7	0.8	0.7	0.4	0.5	0.1	0.1	0.0114	0.0125	0.0002	0.0112	0.0128	0.0002
0.8	0.7	0.8	0.7	0.6	0.7	0.1	0.1	0.0129	0.013	0	0.0116	0.0143	0.0002
0.8	0.7	0.8	0.7	0.8	0.9	0.1	0.1	0.0124	0.0127	0	0.0106	0.0131	0.0002
0.8	0.7	0.8	0.7	0.2	0.1	0.1	0.1	0.0109	0.0124	0.0002	0.0138	0.0132	0.0002
0.8	0.7	0.8	0.7	0.2	0.4	0.1	0.1	0.0127	0.0128	0.0001	0.0127	0.0118	0.0002
0.8	0.7	0.8	0.7	0.2	0.5	0.1	0.1	0.0124	0.0131	0.0001	0.0118	0.0126	0
0.8	0.7	0.8	0.7	0.2	0.3	0.05	0.05	0.0132	0.0138	0.0002	0.0108	0.0135	0.0001
0.8	0.7	0.8	0.7	0.2	0.3	0.15	0.15	0.0148	0.0136	0.0004	0.0146	0.0123	0.0004

Power

Se_C	Sp_C	Se_E	Sp_E	π_{true}	$\pi_{ass.}$	non-inferiority margin sensitivity	non-inferiority margin specificity	Power sensitivity fixed design	Power specificity fixed design	Power overall fixed design	Power sensitivity adaptive design	Power specificitiy adaptive design	Power overall adaptive design
0.2	0.7	0.8	0.7	0.2	0.3	0.1	0.1	1	0.7809	0.7809	1	0.7195	0.7195
0.6	0.7	0.6	0.7	0.2	0.3	0.1	0.1	0.5222	0.9954	0.5196	0.7162	1	0.7162
0.8	0.7	0.8	0.7	0.2	0.3	0.1	0.1	0.5383	0.9724	0.5253	0.7087	0.9958	0.7063
0.8	0.6	0.8	0.6	0.2	0.3	0.1	0.1	0.5553	0.9552	0.5316	0.7095	0.9899	0.7035
0.8	0.8	0.8	0.8	0.2	0.3	0.1	0.1	0.5159	0.9905	0.5111	0.7105	0.9993	0.7101
0.8	0.7	0.8	0.7	0.4	0.5	0.1	0.1	0.7985	0.8653	0.6914	0.801	0.8701	0.6979
0.8	0.7	0.8	0.7	0.6	0.7	0.1	0.1	0.9897	0.8507	0.842	0.9638	0.7354	0.7082
0.8	0.7	0.8	0.7	0.8	0.9	0.1	0.1	1	0.9594	0.9594	1	0.7216	0.7216
0.8	0.7	0.8	0.7	0.2	0.1	0.1	0.1	0.9561	1	0.9561	0.7131	0.9962	0.7108
0.8	0.7	0.8	0.7	0.2	0.4	0.1	0.1	0.4687	0.9494	0.4473	0.7098	0.9967	0.7078
0.8	0.7	0.8	0.7	0.2	0.5	0.1	0.1	0.4671	0.9454	0.4439	0.7114	0.9972	0.7096
0.8	0.7	0.8	0.7	0.2	0.3	0.05	0.05	0.545	0.9707	0.5279	0.7156	0.9957	0.7125
0.8	0.7	0.8	0.7	0.2	0.3	0.15	0.15	0.5337	0.9743	0.5209	0.7054	0.9957	0.7033

Sample size and bias

Se_C	Sp_C	Se_E	Sp_E	π_{true}	$\pi_{ass.}$	non-inferiority margin sensitivity	non-inferiority margin specificity	true sample size	sample size fixed design	sample size interim analysis	sample size adaptive design	$\hat{\pi}$	bias = $\frac{\hat{\pi} - \pi_{true}}{\pi_{true}}$
0.2	0.7	0.8	0.7	0.2	0.3	0.1	0.1	413	472	236	414	0.2000	-0.0002
0.6	0.7	0.6	0.7	0.2	0.3	0.1	0.1	1885	1270	635	1892	0.2001	0.0003
0.8	0.7	0.8	0.7	0.2	0.3	0.1	0.1	1265	894	447	1270	0.2000	0.0002
0.8	0.6	0.8	0.6	0.2	0.3	0.1	0.1	1270	924	462	1276	0.2001	0.0003
0.8	0.8	0.8	0.8	0.2	0.3	0.1	0.1	1268	855	428	1272	0.2000	< 0.001
0.8	0.7	0.8	0.7	0.4	0.5	0.1	0.1	775	770	385	779	0.3997	-0.0007
0.8	0.7	0.8	0.7	0.6	0.7	0.1	0.1	860	1106	553	864	0.6001	0.0001
0.8	0.7	0.8	0.7	0.8	0.9	0.1	0.1	1651	3301	1651	1653	0.8000	< 0.001
0.8	0.7	0.8	0.7	0.2	0.1	0.1	0.1	1265	2520	1260	1267	0.1999	-0.0005
0.8	0.7	0.8	0.7	0.2	0.4	0.1	0.1	1265	775	388	1272	0.2000	-0.0001
0.8	0.7	0.8	0.7	0.2	0.5	0.1	0.1	1265	770	385	1272	0.1999	-0.0003
0.8	0.7	0.8	0.7	0.2	0.3	0.05	0.05	5045	3567	1784	5052	0.2000	0.0001
0.8	0.7	0.8	0.7	0.2	0.3	0.15	0.15	565	398	199	570	0.2001	0.0007
