# Supplementary material for: Blinded sample size re-estimation in a comparative diagnostic accuracy study
Source: BMC Med Res Methodol. 2022 Apr 19;22:115. doi: 10.1186/s12874-022-01564-2 (PMC9019976; doi:10.1186/s12874-022-01564-2)
Supplement: Supplementary file 5 — Additional file 5. Simulation results of the blinded sample size re-estimation in the paired design. [file 12874_2022_1564_MOESM5_ESM.pdf]

Paired design

1. Testing for superiority in sensitivity and specificity (for hypothesis see manuscript, section 2)

Type I error rate

Se_C	Sp_C	Se_E	Sp_E	$\psi_{D_{true}}$	$\psi_{D_{ass.}}$	$\psi_{ND_{true}}$	$\psi_{ND_{ass.}}$	π_{true}	$\pi_{ass.}$	Type I error sensitivity fixed design	Type I error specificity fixed design	Type I error global fixed design	Type I error sensitivity adaptive design	Type I error specificity adaptive design	Type I error global adaptive design
0.81	0.66	0.9	0.8	0.11	0.09	0.14	0.14	0.44	0.47	0.0457	0.0406	0.0017	0.0473	0.047	0.0016
0.8	0.7	0.9	0.8	0.11	0.18	0.14	0.24	0.2	0.3	0.0407	0.0527	0.0015	0.0474	0.0502	0.0024
0.6	0.7	0.7	0.8	0.11	0.18	0.14	0.24	0.2	0.3	0.0446	0.0527	0.0021	0.0477	0.0541	0.0029
0.7	0.7	0.8	0.8	0.11	0.18	0.14	0.24	0.2	0.3	0.0441	0.0527	0.0022	0.0485	0.0516	0.0027
0.8	0.6	0.9	0.7	0.11	0.18	0.14	0.24	0.2	0.3	0.0407	0.0523	0.0018	0.048	0.0526	0.0026
0.8	0.8	0.9	0.9	0.11	0.18	0.14	0.24	0.2	0.3	0.0407	0.049	0.0014	0.0473	0.0481	0.0024
0.8	0.7	0.9	0.8	0.11	0.18	0.14	0.24	0.4	0.5	0.0461	0.0535	0.0023	0.0455	0.0524	0.0021
0.8	0.7	0.9	0.8	0.11	0.18	0.14	0.24	0.6	0.7	0.0495	0.0538	0.0014	0.0491	0.0485	0.0026
0.8	0.7	0.9	0.8	0.11	0.18	0.14	0.24	0.8	0.9	0.0487	0.0523	0.0024	0.0512	0.0487	0.0028
0.8	0.7	0.9	0.8	0.11	0.18	0.14	0.24	0.2	0.1	0.0513	0.0488	0.0026	0.0413	0.0506	0.0025
0.8	0.7	0.9	0.8	0.11	0.18	0.14	0.24	0.2	0.4	0.0428	0.0518	0.0018	0.0461	0.0515	0.0031
0.8	0.7	0.9	0.8	0.11	0.18	0.14	0.24	0.2	0.5	0.043	0.0517	0.0017	0.0481	0.053	0.0025
0.8	0.7	0.9	0.8	0.18	0.18	0.14	0.24	0.2	0.3	0.0478	0.0527	0.0013	0.0508	0.0496	0.0017
0.8	0.7	0.9	0.8	0.26	0.18	0.14	0.24	0.2	0.3	0.0556	0.0527	0.0017	0.0495	0.0476	0.0027
0.8	0.7	0.9	0.8	0.11	0.18	0.24	0.24	0.2	0.3	0.0407	0.0535	0.0021	0.0471	0.0505	0.0026
0.8	0.7	0.9	0.8	0.11	0.18	0.38	0.24	0.2	0.3	0.0407	0.0529	0.0016	0.044	0.0511	0.0021
0.8	0.7	0.85	0.8	0.11	0.18	0.14	0.24	0.2	0.3	0.0475	0.0507	0.0023	0.0524	0.0488	0.0022
0.8	0.7	0.95	0.8	0.15	0.18	0.14	0.24	0.2	0.3	0.0444	0.0509	0.0031	0.0428	0.0516	0.0022
0.8	0.7	0.9	0.75	0.11	0.18	0.14	0.24	0.2	0.3	0.0495	0.0499	0.0028	0.0413	0.051	0.0021
0.8	0.7	0.9	0.85	0.11	0.18	0.15	0.24	0.2	0.3	0.0426	0.0497	0.002	0.0451	0.0506	0.0019

Power

Se_C	Sp_C	Se_E	Sp_E	$\psi_{D_{true}}$	$\psi_{D_{ass.}}$	$\psi_{ND_{true}}$	$\psi_{ND_{ass.}}$	π_{true}	$\pi_{ass.}$	Power sensitivity fixed design	Power specificity fixed design	Power overall fixed design	Power sensitivity adaptive design	Power specificitiy adaptive design	Power overall adaptive design
0.81	0.66	0.9	0.8	0.11	0.09	0.14	0.14	0.44	0.47	0.6122	0.9939	0.6077	0.7637	0.9982	0.7626
0.8	0.7	0.9	0.8	0.11	0.18	0.14	0.24	0.2	0.3	0.9176	0.9999	0.9175	0.7855	0.9944	0.783
0.6	0.7	0.7	0.8	0.11	0.18	0.14	0.24	0.2	0.3	0.9429	0.9999	0.9428	0.8032	0.9941	0.799
0.7	0.7	0.8	0.8	0.11	0.18	0.14	0.24	0.2	0.3	0.9402	0.9999	0.9401	0.7998	0.9943	0.796
0.8	0.6	0.9	0.7	0.11	0.18	0.14	0.24	0.2	0.3	0.9176	0.9997	0.9173	0.7867	0.9938	0.7828
0.8	0.8	0.9	0.9	0.11	0.18	0.14	0.24	0.2	0.3	0.9176	0.9999	0.9176	0.7857	0.9946	0.7821
0.8	0.7	0.9	0.8	0.11	0.18	0.14	0.24	0.4	0.5	0.9937	0.9952	0.9889	0.8575	0.8742	0.7639
0.8	0.7	0.9	0.8	0.11	0.18	0.14	0.24	0.6	0.7	1	0.9946	0.9946	0.9793	0.7631	0.7516
0.8	0.7	0.9	0.8	0.11	0.18	0.14	0.24	0.8	0.9	1	0.9999	0.9999	1	0.8546	0.8546
0.8	0.7	0.9	0.8	0.11	0.18	0.14	0.24	0.2	0.1	0.9999	1	0.9999	0.958	0.9999	0.9579
0.8	0.7	0.9	0.8	0.11	0.18	0.14	0.24	0.2	0.4	0.8749	0.9999	0.8748	0.7868	0.9945	0.7835
0.8	0.7	0.9	0.8	0.11	0.18	0.14	0.24	0.2	0.5	0.8754	0.9999	0.8753	0.7892	0.995	0.7863
0.8	0.7	0.9	0.8	0.18	0.18	0.14	0.24	0.2	0.3	0.6416	0.9999	0.6415	0.7345	0.9982	0.734
0.8	0.7	0.9	0.8	0.26	0.18	0.14	0.24	0.2	0.3	0.4872	0.9999	0.4872	0.7595	0.9998	0.7595
0.8	0.7	0.9	0.8	0.11	0.18	0.24	0.24	0.2	0.3	0.9176	0.9807	0.8996	0.826	0.9436	0.785
0.8	0.7	0.9	0.8	0.11	0.18	0.38	0.24	0.2	0.3	0.9176	0.8802	0.8073	0.8941	0.8801	0.7921
0.8	0.7	0.85	0.8	0.11	0.18	0.14	0.24	0.2	0.3	0.8404	1	0.8404	0.7397	1	0.7397
0.8	0.7	0.95	0.8	0.15	0.18	0.14	0.24	0.2	0.3	0.9642	0.9923	0.9566	0.8694	0.9656	0.8435
0.8	0.7	0.9	0.75	0.11	0.18	0.14	0.24	0.2	0.3	0.9993	0.9792	0.9786	0.9497	0.8261	0.7887
0.8	0.7	0.9	0.85	0.11	0.18	0.15	0.24	0.2	0.3	0.9005	1	0.9005	0.7818	1	0.7818

Sample size and bias under the alternative hypothesis

Se_C	Sp_C	Se_E	Sp_E	$\psi_{D_{true}}$	$\psi_{D_{ass.}}$	$\psi_{ND_{true}}$	$\psi_{ND_{ass.}}$	π_{true}	$\pi_{ass.}$	true sample size	sample size fixed design	sample size interim analysis	sample size adaptive design	$\hat{\pi}$	bias = $\frac{\hat{\pi}-\pi_{true}}{\pi_{true}}$	$\hat{\psi}_D$	bias = $\frac{\hat{\psi}_D-\psi_{D_{true}}}{\psi_{D_{true}}}$	$\hat{\psi}_{ND}$	bias = $\frac{\hat{\psi}_{ND}-\psi_{ND_{true}}}{\psi_{ND_{true}}}$
0.81	0.66	0.9	0.8	0.11	0.09	0.14	0.14	0.44	0.47	200	133	133	222	0.44	0.0001	0.1177	0.0702	0.1558	0.1131
0.8	0.7	0.9	0.8	0.11	0.18	0.14	0.24	0.2	0.3	336	463	187	416	0.1997	-0.0015	0.1258	0.1436	0.1407	0.005
0.6	0.7	0.7	0.8	0.11	0.18	0.14	0.24	0.2	0.3	336	463	187	404	0.1997	-0.0015	0.1234	0.1214	0.1407	0.005
0.7	0.7	0.8	0.8	0.11	0.18	0.14	0.24	0.2	0.3	336	463	187	403	0.1997	-0.0015	0.1232	0.1202	0.1407	0.005
0.8	0.6	0.9	0.7	0.11	0.18	0.14	0.24	0.2	0.3	336	463	187	416	0.1997	-0.0015	0.1258	0.1436	0.1415	0.0107
0.8	0.8	0.9	0.9	0.11	0.18	0.14	0.24	0.2	0.3	336	463	187	416	0.1997	-0.0015	0.1258	0.1436	0.1405	0.0036
0.8	0.7	0.9	0.8	0.11	0.18	0.14	0.24	0.4	0.5	201	405	131	233	0.4002	0.0005	0.1224	0.1129	0.1425	0.0175
0.8	0.7	0.9	0.8	0.11	0.18	0.14	0.24	0.6	0.7	240	602	186	254	0.6005	0.0008	0.1175	0.0682	0.1423	0.0165
0.8	0.7	0.9	0.8	0.11	0.18	0.14	0.24	0.8	0.9	478	1803	557	482	0.8001	0.0001	0.1121	0.0192	0.1411	0.008
0.8	0.7	0.9	0.8	0.11	0.18	0.14	0.24	0.2	0.1	336	1304	557	371	0.2001	0.0006	0.1175	0.0678	0.1395	-0.0038
0.8	0.7	0.9	0.8	0.11	0.18	0.14	0.24	0.2	0.4	336	404	143	436	0.1998	-0.0009	0.1295	0.177	0.1415	0.0104
0.8	0.7	0.9	0.8	0.11	0.18	0.14	0.24	0.2	0.5	336	405	131	439	0.2	-0.0002	0.1299	0.1809	0.1419	0.0134
0.8	0.7	0.9	0.8	0.18	0.18	0.14	0.24	0.2	0.3	652	463	187	672	0.1997	-0.0015	0.1826	0.0147	0.1407	0.005
0.8	0.7	0.9	0.8	0.26	0.18	0.14	0.24	0.2	0.3	983	463	187	949	0.1997	-0.0015	0.247	-0.05	0.1407	0.005
0.8	0.7	0.9	0.8	0.11	0.18	0.24	0.24	0.2	0.3	362	463	187	439	0.1997	-0.0015	0.1258	0.1436	0.2414	0.006
0.8	0.7	0.9	0.8	0.11	0.18	0.38	0.24	0.2	0.3	437	463	187	506	0.1997	-0.0015	0.1258	0.1436	0.3806	0.0016
0.8	0.7	0.85	0.8	0.11	0.18	0.14	0.24	0.2	0.3	1642	1850	375	1656	0.2	0.0001	0.1105	0.0048	0.1396	-0.003
0.8	0.7	0.95	0.8	0.15	0.18	0.14	0.24	0.2	0.3	193	275	126	259	0.2	< 0.0001	0.1752	0.1682	0.1413	0.0093
0.8	0.7	0.9	0.75	0.11	0.18	0.14	0.24	0.2	0.3	556	1075	205	617	0.2	0.0002	0.1243	0.1301	0.1399	-0.0007
0.8	0.7	0.9	0.85	0.11	0.18	0.15	0.24	0.2	0.3	334	435	186	413	0.1998	-0.0009	0.1257	0.1426	0.1623	0.0822

2. Testing for superiority in sensitivity and non-inferiority in specificity (for hypothesis see Additional file 1, section A.I)

Type I error rate

Se_C	Sp_C	Se_E	Sp_E	$\psi_{D_{true}}$	$\psi_{D_{ass.}}$	$\psi_{ND_{true}}$	$\psi_{ND_{ass.}}$	π_{true}	$\pi_{ass.}$	Non-inferiority margin specificity	Type I error sensitivity fixed design	Type I error specificity fixed design	Type I error global fixed design	Type I error sensitivity adaptive design	Type I error specificity adaptive design	Type I error global adaptive design
0.8	0.7	0.9	0.7	0.11	0.18	0.14	0.24	0.2	0.3	0.1	0.042	0.0257	0.0011	0.0441	0.023	0.0011
0.6	0.7	0.7	0.7	0.11	0.18	0.14	0.24	0.2	0.3	0.1	0.0443	0.0257	0.0013	0.0494	0.0238	0.001
0.7	0.7	0.8	0.7	0.11	0.18	0.14	0.24	0.2	0.3	0.1	0.0434	0.0257	0.0009	0.0492	0.0247	0.0011
0.8	0.6	0.9	0.6	0.11	0.18	0.14	0.24	0.2	0.3	0.1	0.042	0.0257	0.0008	0.0438	0.0236	0.0011
0.8	0.8	0.9	0.8	0.11	0.18	0.14	0.24	0.2	0.3	0.1	0.042	0.0212	0.0013	0.045	0.0224	0.0014
0.8	0.7	0.9	0.7	0.11	0.18	0.14	0.24	0.4	0.5	0.1	0.0473	0.0208	0.0012	0.0426	0.0214	0.0005
0.8	0.7	0.9	0.7	0.11	0.18	0.14	0.24	0.6	0.7	0.1	0.0492	0.0218	0.0006	0.0523	0.0246	0.0011
0.8	0.7	0.9	0.7	0.11	0.18	0.14	0.24	0.8	0.9	0.1	0.0513	0.0247	0.0016	0.0486	0.0227	0.0017
0.8	0.7	0.9	0.7	0.11	0.18	0.14	0.24	0.2	0.1	0.1	0.0513	0.0235	0.0012	0.0413	0.0237	0.0008
0.8	0.7	0.9	0.7	0.11	0.18	0.14	0.24	0.2	0.4	0.1	0.0422	0.0235	0.0014	0.048	0.0246	0.0013
0.8	0.7	0.9	0.7	0.11	0.18	0.14	0.24	0.2	0.5	0.1	0.0425	0.0237	0.0011	0.0468	0.0239	0.0014
0.8	0.7	0.9	0.7	0.18	0.18	0.14	0.24	0.2	0.3	0.1	0.0494	0.0257	0.0013	0.049	0.0258	0.0013
0.8	0.7	0.9	0.7	0.26	0.18	0.14	0.24	0.2	0.3	0.1	0.0558	0.0257	0.0018	0.0462	0.0243	0.0008
0.8	0.7	0.9	0.7	0.11	0.18	0.21	0.24	0.2	0.3	0.1	0.042	0.027	0.0014	0.0452	0.0259	0.001
0.8	0.7	0.9	0.7	0.11	0.18	0.42	0.24	0.2	0.3	0.1	0.042	0.0257	0.0011	0.0456	0.026	0.0015
0.8	0.7	0.85	0.7	0.11	0.18	0.14	0.24	0.2	0.3	0.1	0.0475	0.0264	0.0011	0.0524	0.0234	0.0013
0.8	0.7	0.95	0.7	0.15	0.18	0.14	0.24	0.2	0.3	0.1	0.0423	0.023	0.0009	0.0438	0.0247	0.0009
0.8	0.7	0.9	0.7	0.11	0.18	0.14	0.24	0.2	0.3	0.05	0.0501	0.0259	0.0011	0.0482	0.0246	0.001
0.8	0.7	0.9	0.7	0.11	0.18	0.14	0.24	0.2	0.3	0.15	0.0431	0.0241	0.0008	0.0497	0.0221	0.001

Power

Se_C	Sp_C	Se_E	Sp_E	$\psi_{D_{true}}$	$\psi_{D_{ass.}}$	$\psi_{ND_{true}}$	$\psi_{ND_{ass.}}$	π_{true}	$\pi_{ass.}$	Non-inferiority margin specificity	Power sensitivity fixed design	Power specificity fixed design	Power overall fixed design	Power sensitivity adaptive design	Power specificitiy adaptive design	Power overall adaptive design
0.8	0.7	0.9	0.7	0.11	0.18	0.14	0.24	0.2	0.3	0.1	0.9241	0.9985	0.9227	0.7977	0.9855	0.7878
0.6	0.7	0.7	0.7	0.11	0.18	0.14	0.24	0.2	0.3	0.1	0.9476	0.9985	0.9462	0.8199	0.985	0.8087
0.7	0.7	0.8	0.7	0.11	0.18	0.14	0.24	0.2	0.3	0.1	0.9464	0.9985	0.945	0.8196	0.9846	0.8089
0.8	0.6	0.9	0.6	0.11	0.18	0.14	0.24	0.2	0.3	0.1	0.9241	0.9989	0.923	0.7976	0.9853	0.7882
0.8	0.8	0.9	0.8	0.11	0.18	0.14	0.24	0.2	0.3	0.1	0.9241	0.9989	0.923	0.7985	0.9862	0.7885
0.8	0.7	0.9	0.7	0.11	0.18	0.14	0.24	0.4	0.5	0.1	0.9961	0.983	0.9792	0.9169	0.8825	0.8102
0.8	0.7	0.9	0.7	0.11	0.18	0.14	0.24	0.6	0.7	0.1	1	0.9847	0.9847	0.9949	0.8298	0.8259
0.8	0.7	0.9	0.7	0.11	0.18	0.14	0.24	0.8	0.9	0.1	1	0.9989	0.9989	1	0.8287	0.8287
0.8	0.7	0.9	0.7	0.11	0.18	0.14	0.24	0.2	0.1	0.1	0.9999	1	0.9999	0.958	0.9997	0.9577
0.8	0.7	0.9	0.7	0.11	0.18	0.14	0.24	0.2	0.4	0.1	0.894	0.9965	0.891	0.8045	0.9858	0.7948
0.8	0.7	0.9	0.7	0.11	0.18	0.14	0.24	0.2	0.5	0.1	0.9005	0.9973	0.8981	0.8023	0.9848	0.7916
0.8	0.7	0.9	0.7	0.18	0.18	0.14	0.24	0.2	0.3	0.1	0.6536	0.9985	0.6529	0.735	0.9967	0.7343
0.8	0.7	0.9	0.7	0.26	0.18	0.14	0.24	0.2	0.3	0.1	0.4976	0.9985	0.4967	0.7556	0.9997	0.7556
0.8	0.7	0.9	0.7	0.11	0.18	0.21	0.24	0.2	0.3	0.1	0.9241	0.9867	0.9115	0.8253	0.9579	0.795
0.8	0.7	0.9	0.7	0.11	0.18	0.42	0.24	0.2	0.3	0.1	0.9241	0.8506	0.7861	0.9203	0.869	0.803
0.8	0.7	0.85	0.7	0.11	0.18	0.14	0.24	0.2	0.3	0.1	0.8404	1	0.8404	0.7397	1	0.7397
0.8	0.7	0.95	0.7	0.15	0.18	0.14	0.24	0.2	0.3	0.1	0.9776	0.975	0.9528	0.8995	0.9479	0.8529
0.8	0.7	0.9	0.7	0.11	0.18	0.14	0.24	0.2	0.3	0.05	0.9995	0.9741	0.9736	0.9603	0.8412	0.8103
0.8	0.7	0.9	0.7	0.11	0.18	0.14	0.24	0.2	0.3	0.15	0.901	1	0.901	0.7755	0.9999	0.7755

Sample size and bias under the alternative hypothesis

Se_C	Sp_C	Se_E	Sp_E	$\psi_{D_{true}}$	$\psi_{D_{ass.}}$	$\psi_{ND_{true}}$	$\psi_{ND_{ass.}}$	π_{true}	$\pi_{ass.}$	Non-inferiority margin specificity	true sample size	sample size fixed design	sample size interim analysis	sample size adaptive design	$\hat{\pi}$	bias = $\frac{\hat{\pi} - \pi_{true}}{\pi_{true}}$	$\hat{\psi}_D$	bias = $\frac{\hat{\psi}_D - \psi_{D_{true}}}{\psi_{D_{true}}}$	$\hat{\psi}_{ND}$	bias = $\frac{\hat{\psi}_{ND} - \psi_{ND_{true}}}{\psi_{ND_{true}}}$
0.8	0.7	0.9	0.7	0.11	0.18	0.14	0.24	0.2	0.3	0.1	341	476	186	422	0.1998	-0.0011	0.1259	0.1446	0.1404	0.0032
0.6	0.7	0.7	0.7	0.11	0.18	0.14	0.24	0.2	0.3	0.1	341	476	186	411	0.1998	-0.0011	0.1237	0.1243	0.1404	0.0032
0.7	0.7	0.8	0.7	0.11	0.18	0.14	0.24	0.2	0.3	0.1	341	476	186	411	0.1998	-0.0011	0.1236	0.1234	0.1404	0.0032
0.8	0.6	0.9	0.6	0.11	0.18	0.14	0.24	0.2	0.3	0.1	341	476	186	422	0.1998	-0.0011	0.1259	0.1446	0.1397	-0.0023
0.8	0.8	0.9	0.8	0.11	0.18	0.14	0.24	0.2	0.3	0.1	341	476	186	422	0.1998	-0.0011	0.1259	0.1446	0.14	< 0.0001
0.8	0.7	0.9	0.7	0.11	0.18	0.14	0.24	0.4	0.5	0.1	239	434	119	270	0.3993	-0.0017	0.1232	0.1204	0.1403	0.002
0.8	0.7	0.9	0.7	0.11	0.18	0.14	0.24	0.6	0.7	0.1	324	662	158	335	0.5998	-0.0003	0.1183	0.0756	0.14	0.0003
0.8	0.7	0.9	0.7	0.11	0.18	0.14	0.24	0.8	0.9	0.1	648	1985	473	658	0.8001	0.0001	0.1126	0.0238	0.1405	0.0036
0.8	0.7	0.9	0.7	0.11	0.18	0.14	0.24	0.2	0.1	0.1	341	1304	557	376	0.2001	0.0006	0.1175	0.0678	0.1403	0.0021
0.8	0.7	0.9	0.7	0.11	0.18	0.14	0.24	0.2	0.4	0.1	341	425	140	436	0.1999	-0.0006	0.1284	0.167	0.141	0.0069
0.8	0.7	0.9	0.7	0.11	0.18	0.14	0.24	0.2	0.5	0.1	341	434	119	446	0.2	< 0.0001	0.1298	0.1804	0.1416	0.0111
0.8	0.7	0.9	0.7	0.18	0.18	0.14	0.24	0.2	0.3	0.1	652	476	186	671	0.1998	-0.0011	0.182	0.0112	0.1404	0.0032
0.8	0.7	0.9	0.7	0.26	0.18	0.14	0.24	0.2	0.3	0.1	983	476	186	944	0.1998	-0.0011	0.2459	-0.0542	0.1404	0.0032
0.8	0.7	0.9	0.7	0.11	0.18	0.21	0.24	0.2	0.3	0.1	361	476	186	438	0.1998	-0.0011	0.1259	0.1446	0.2096	-0.0018
0.8	0.7	0.9	0.7	0.11	0.18	0.42	0.24	0.2	0.3	0.1	469	476	186	538	0.1998	-0.0011	0.1259	0.1446	0.421	0.0024
0.8	0.7	0.85	0.7	0.11	0.18	0.14	0.24	0.2	0.3	0.1	1642	1850	375	1656	0.2	0.0001	0.1105	0.0048	0.1403	0.002
0.8	0.7	0.95	0.7	0.15	0.18	0.14	0.24	0.2	0.3	0.1	213	296	123	273	0.2002	0.0011	0.1754	0.1692	0.1398	-0.0012
0.8	0.7	0.9	0.7	0.11	0.18	0.14	0.24	0.2	0.3	0.05	597	1101	204	659	0.1998	-0.0009	0.1249	0.1355	0.1402	0.0013
0.8	0.7	0.9	0.7	0.11	0.18	0.14	0.24	0.2	0.3	0.15	334	436	186	414	0.1998	-0.001	0.1259	0.1442	0.1408	0.0059

3. Testing for non-inferiority in sensitivity and superiority in specificity (for hypothesis see Additional file 1, section A.II)

Type I error rate

Se_C	Sp_C	Se_E	Sp_E	$\psi_{D_{true}}$	$\psi_{D_{ass.}}$	$\psi_{ND_{true}}$	$\psi_{ND_{ass.}}$	π_{true}	$\pi_{ass.}$	Non-inferiority margin sensitivity	Type I error sensitivity fixed design	Type I error specificity fixed design	Type I error global fixed design	Type I error sensitivity adaptive design	Type I error specificity adaptive design	Type I error global adaptive design
0.8	0.7	0.8	0.8	0.11	0.18	0.14	0.24	0.2	0.3	0.1	0.0159	0.0531	0.0011	0.0188	0.0491	0.0009
0.6	0.7	0.6	0.8	0.11	0.18	0.14	0.24	0.2	0.3	0.1	0.0204	0.0531	0.0009	0.0229	0.0513	0.0012
0.7	0.7	0.7	0.8	0.11	0.18	0.14	0.24	0.2	0.3	0.1	0.0161	0.0531	0.0007	0.0186	0.0511	0.0008
0.8	0.6	0.8	0.7	0.11	0.18	0.14	0.24	0.2	0.3	0.1	0.0159	0.0512	0.0013	0.0185	0.0542	0.001
0.8	0.8	0.8	0.9	0.11	0.18	0.14	0.24	0.2	0.3	0.1	0.0159	0.0475	0.0007	0.019	0.0514	0.0005
0.8	0.7	0.8	0.8	0.11	0.18	0.14	0.24	0.4	0.5	0.1	0.018	0.0533	0.0007	0.0183	0.0512	0.0005
0.8	0.7	0.8	0.8	0.11	0.18	0.14	0.24	0.6	0.7	0.1	0.0201	0.0536	0.0008	0.0178	0.0448	0.0004
0.8	0.7	0.8	0.8	0.11	0.18	0.14	0.24	0.8	0.9	0.1	0.0207	0.0523	0.0011	0.0216	0.0487	0.0011
0.8	0.7	0.8	0.8	0.11	0.18	0.14	0.24	0.2	0.1	0.1	0.0202	0.0484	0.0007	0.0191	0.048	0.0009
0.8	0.7	0.8	0.8	0.11	0.18	0.14	0.24	0.2	0.4	0.1	0.0167	0.0527	0.0012	0.0198	0.0512	0.0014
0.8	0.7	0.8	0.8	0.11	0.18	0.14	0.24	0.2	0.5	0.1	0.017	0.0524	0.0013	0.0193	0.0499	0.0023
0.8	0.7	0.8	0.8	0.16	0.18	0.14	0.24	0.2	0.3	0.1	0.0236	0.0531	0.0013	0.0246	0.0482	0.0014
0.8	0.7	0.8	0.8	0.32	0.18	0.14	0.24	0.2	0.3	0.1	0.0254	0.0531	0.0017	0.025	0.0482	0.0013
0.8	0.7	0.8	0.8	0.11	0.18	0.24	0.24	0.2	0.3	0.1	0.0159	0.0491	0.0009	0.0178	0.0503	0.0009
0.8	0.7	0.8	0.8	0.11	0.18	0.38	0.24	0.2	0.3	0.1	0.0159	0.0505	0.0006	0.0187	0.0487	0.0008
0.8	0.7	0.8	0.8	0.11	0.18	0.14	0.24	0.2	0.3	0.05	0.0224	0.0501	0.0009	0.0243	0.0504	0.0015
0.8	0.7	0.8	0.8	0.11	0.18	0.14	0.24	0.2	0.3	0.15	0.0172	0.0531	0.0012	0.0192	0.0511	0.0011
0.8	0.7	0.8	0.75	0.11	0.18	0.14	0.24	0.2	0.3	0.1	0.0204	0.0507	0.0015	0.0189	0.0493	0.0008
0.8	0.7	0.8	0.85	0.11	0.18	0.15	0.24	0.2	0.3	0.1	0.0179	0.0513	0.0014	0.0189	0.0521	0.0009

Power

Se_C	Sp_C	Se_E	Sp_E	$\psi_{D_{true}}$	$\psi_{D_{ass.}}$	$\psi_{ND_{true}}$	$\psi_{ND_{ass.}}$	π_{true}	$\pi_{ass.}$	Non-inferiority margin sensitivity	Power sensitivity fixed design	Power specificity fixed design	Power overall fixed design	Power sensitivity adaptive design	Power specificitiy adaptive design	Power overall adaptive design
0.8	0.7	0.8	0.8	0.11	0.18	0.14	0.24	0.2	0.3	0.1	0.8146	1	0.8146	0.8225	0.9986	0.8215
0.6	0.7	0.6	0.8	0.11	0.18	0.14	0.24	0.2	0.3	0.1	0.8163	1	0.8163	0.8132	0.9989	0.8126
0.7	0.7	0.7	0.8	0.11	0.18	0.14	0.24	0.2	0.3	0.1	0.8116	1	0.8116	0.8094	0.9987	0.8088
0.8	0.6	0.8	0.7	0.11	0.18	0.14	0.24	0.2	0.3	0.1	0.8146	0.9999	0.8145	0.8235	0.9991	0.8229
0.8	0.8	0.8	0.9	0.11	0.18	0.14	0.24	0.2	0.3	0.1	0.8146	1	0.8146	0.8241	0.9991	0.8235
0.8	0.7	0.8	0.8	0.11	0.18	0.14	0.24	0.4	0.5	0.1	0.9595	0.9974	0.9571	0.859	0.9529	0.8223
0.8	0.7	0.8	0.8	0.11	0.18	0.14	0.24	0.6	0.7	0.1	0.9998	0.9954	0.9952	0.9367	0.8023	0.7555
0.8	0.7	0.8	0.8	0.11	0.18	0.14	0.24	0.8	0.9	0.1	1	0.9999	0.9999	1	0.8547	0.8547
0.8	0.7	0.8	0.8	0.11	0.18	0.14	0.24	0.2	0.1	0.1	0.9991	1	0.9991	0.842	1	0.842
0.8	0.7	0.8	0.8	0.11	0.18	0.14	0.24	0.2	0.4	0.1	0.7339	0.9999	0.7339	0.8046	0.9983	0.8035
0.8	0.7	0.8	0.8	0.11	0.18	0.14	0.24	0.2	0.5	0.1	0.7135	1	0.7135	0.7994	0.9974	0.7977
0.8	0.7	0.8	0.8	0.16	0.18	0.14	0.24	0.2	0.3	0.1	0.6859	1	0.6859	0.8046	0.9996	0.8045
0.8	0.7	0.8	0.8	0.32	0.18	0.14	0.24	0.2	0.3	0.1	0.4387	1	0.4387	0.7889	1	0.7889
0.8	0.7	0.8	0.8	0.11	0.18	0.24	0.24	0.2	0.3	0.1	0.8146	0.9917	0.8077	0.8347	0.9793	0.8188
0.8	0.7	0.8	0.8	0.11	0.18	0.38	0.24	0.2	0.3	0.1	0.8146	0.9231	0.7523	0.87	0.9365	0.8171
0.8	0.7	0.8	0.8	0.11	0.18	0.14	0.24	0.2	0.3	0.05	0.816	1	0.816	0.7874	1	0.7874
0.8	0.7	0.8	0.8	0.11	0.18	0.14	0.24	0.2	0.3	0.15	0.8863	0.9976	0.8841	0.8395	0.977	0.8217
0.8	0.7	0.8	0.75	0.11	0.18	0.14	0.24	0.2	0.3	0.1	0.9889	0.9809	0.97	0.9103	0.8817	0.8049
0.8	0.7	0.8	0.85	0.11	0.18	0.15	0.24	0.2	0.3	0.1	0.7981	1	0.7981	0.8137	1	0.8137

Sample size and bias under alternative hypothesis

Se_C	Sp_C	Se_E	Sp_E	$\psi_{D_{true}}$	$\psi_{D_{ass.}}$	$\psi_{ND_{true}}$	$\psi_{ND_{ass.}}$	π_{true}	$\pi_{ass.}$	Non-inferiority margin sensitivity	true sample size	sample size fixed design	sample size interim analysis	sample size adaptive design	$\hat{\pi}$	bias = $\frac{\hat{\pi}-\pi_{true}}{\pi_{true}}$	$\hat{\psi}_D$	bias = $\frac{\hat{\psi}_D-\psi_{D_{true}}}{\psi_{D_{true}}}$	$\hat{\psi}_{ND}$	bias = $\frac{\hat{\psi}_{ND}-\psi_{ND_{true}}}{\psi_{ND_{true}}}$
0.8	0.7	0.8	0.8	0.11	0.18	0.14	0.24	0.2	0.3	0.1	551	538	158	578	0.1997	-0.0014	0.1115	0.0137	0.1403	0.0024
0.6	0.7	0.6	0.8	0.11	0.18	0.14	0.24	0.2	0.3	0.1	551	538	158	574	0.1997	-0.0014	0.1102	0.0021	0.1403	0.0024
0.7	0.7	0.7	0.8	0.11	0.18	0.14	0.24	0.2	0.3	0.1	551	538	158	576	0.1997	-0.0014	0.1107	0.0061	0.1403	0.0024
0.8	0.6	0.8	0.7	0.11	0.18	0.14	0.24	0.2	0.3	0.1	551	538	158	579	0.1997	-0.0014	0.1115	0.0137	0.1421	0.015
0.8	0.8	0.8	0.9	0.11	0.18	0.14	0.24	0.2	0.3	0.1	551	538	158	578	0.1997	-0.0014	0.1115	0.0137	0.1406	0.0041
0.8	0.7	0.8	0.8	0.11	0.18	0.14	0.24	0.4	0.5	0.1	288	438	119	306	0.3995	-0.0014	0.111	0.0087	0.1431	0.0219
0.8	0.7	0.8	0.8	0.11	0.18	0.14	0.24	0.6	0.7	0.1	271	606	186	280	0.6005	0.0009	0.1105	0.0046	0.1423	0.0165
0.8	0.7	0.8	0.8	0.11	0.18	0.14	0.24	0.8	0.9	0.1	478	1803	557	483	0.8001	0.0001	0.1104	0.004	0.1411	0.008
0.8	0.7	0.8	0.8	0.11	0.18	0.14	0.24	0.2	0.1	0.1	551	1564	473	561	0.1999	-0.0003	0.1107	0.0064	0.1394	-0.0046
0.8	0.7	0.8	0.8	0.11	0.18	0.14	0.24	0.2	0.4	0.1	551	452	125	582	0.1996	-0.0021	0.1105	0.005	0.1418	0.0128
0.8	0.7	0.8	0.8	0.11	0.18	0.14	0.24	0.2	0.5	0.1	551	438	119	583	0.1998	-0.001	0.1108	0.0077	0.1426	0.0184
0.8	0.7	0.8	0.8	0.16	0.18	0.14	0.24	0.2	0.3	0.1	714	538	158	745	0.1997	-0.0014	0.1606	0.0038	0.1403	0.0024
0.8	0.7	0.8	0.8	0.32	0.18	0.14	0.24	0.2	0.3	0.1	1284	538	158	1294	0.1997	-0.0014	0.3122	-0.0245	0.1403	0.0024
0.8	0.7	0.8	0.8	0.11	0.18	0.24	0.24	0.2	0.3	0.1	556	538	158	591	0.1997	-0.0014	0.1115	0.0137	0.2409	0.0039
0.8	0.7	0.8	0.8	0.11	0.18	0.38	0.24	0.2	0.3	0.1	606	538	158	640	0.1997	-0.0014	0.1115	0.0137	0.3802	0.0006
0.8	0.7	0.8	0.8	0.11	0.18	0.14	0.24	0.2	0.3	0.05	1890	1945	380	1914	0.2002	0.0008	0.1095	-0.0045	0.1394	-0.0039
0.8	0.7	0.8	0.8	0.11	0.18	0.14	0.24	0.2	0.3	0.15	282	328	100	305	0.1994	-0.0029	0.1113	0.0121	0.1423	0.0162
0.8	0.7	0.8	0.75	0.11	0.18	0.14	0.24	0.2	0.3	0.1	698	1099	182	725	0.1999	-0.0003	0.1099	-0.0011	0.1398	-0.0017
0.8	0.7	0.8	0.85	0.11	0.18	0.15	0.24	0.2	0.3	0.1	551	522	158	575	0.1994	-0.0029	0.1108	0.0077	0.1625	0.0835

4. Testing for non-inferiority in sensitivity and specificity (for hypothesis see Additional file 1, section A.III)

Type I error rate

Se_C	Sp_C	Se_E	Sp_E	$\psi_{D_{true}}$	$\psi_{D_{ass.}}$	$\psi_{ND_{true}}$	$\psi_{ND_{ass.}}$	π_{true}	$\pi_{ass.}$	Non-inferiority margin sensitivity	Non-inferiority margin specificity	Type I error sensitivity fixed design	Type I error specificity fixed design	Type I error global fixed design	Type I error sensitivity adaptive design	Type I error specificity adaptive design	Type I error global adaptive design
0.8	0.7	0.8	0.7	0.11	0.18	0.14	0.24	0.2	0.3	0.1	0.1	0.0158	0.0236	0.0004	0.0206	0.0227	0.0003
0.6	0.7	0.6	0.7	0.11	0.18	0.14	0.24	0.2	0.3	0.1	0.1	0.0194	0.0236	0.0007	0.0197	0.0223	0.0004
0.8	0.7	0.8	0.7	0.11	0.18	0.14	0.24	0.2	0.3	0.1	0.1	0.0158	0.0236	0.0004	0.0206	0.0227	0.0003
0.8	0.6	0.8	0.6	0.11	0.18	0.14	0.24	0.2	0.3	0.1	0.1	0.0158	0.0265	0.0006	0.0212	0.0265	0.0007
0.8	0.8	0.8	0.8	0.11	0.18	0.14	0.24	0.2	0.3	0.1	0.1	0.0158	0.0201	0.0003	0.0206	0.0207	0.0006
0.8	0.7	0.8	0.7	0.11	0.18	0.14	0.24	0.4	0.5	0.1	0.1	0.0181	0.0243	0.0006	0.0197	0.0205	0.0003
0.8	0.7	0.8	0.7	0.11	0.18	0.14	0.24	0.6	0.7	0.1	0.1	0.0201	0.0219	0.0009	0.0198	0.0238	0.0004
0.8	0.7	0.8	0.7	0.11	0.18	0.14	0.24	0.8	0.9	0.1	0.1	0.0251	0.0247	0.0007	0.0214	0.0226	0.0009
0.8	0.7	0.8	0.7	0.11	0.18	0.14	0.24	0.2	0.1	0.1	0.1	0.0202	0.0242	0.0005	0.0191	0.0247	0.0002
0.8	0.7	0.8	0.7	0.11	0.18	0.14	0.24	0.2	0.4	0.1	0.1	0.0166	0.0242	0.0002	0.0207	0.0244	0.0005
0.8	0.7	0.8	0.7	0.11	0.18	0.14	0.24	0.2	0.5	0.1	0.1	0.017	0.0242	0.0004	0.0185	0.0224	0.0005
0.8	0.7	0.8	0.7	0.16	0.18	0.14	0.24	0.2	0.3	0.1	0.1	0.0243	0.0236	0.0007	0.0265	0.0233	0.0007
0.8	0.7	0.8	0.7	0.32	0.18	0.14	0.24	0.2	0.3	0.1	0.1	0.0245	0.0236	0.001	0.0226	0.0258	0.0009
0.8	0.7	0.8	0.7	0.11	0.18	0.21	0.24	0.2	0.3	0.1	0.1	0.0158	0.0261	0.0005	0.0219	0.0244	0.0002
0.8	0.7	0.8	0.7	0.11	0.18	0.42	0.24	0.2	0.3	0.1	0.1	0.0158	0.0245	0.0005	0.018	0.0211	0.0003
0.8	0.7	0.8	0.7	0.11	0.18	0.14	0.24	0.2	0.3	0.05	0.05	0.0231	0.0268	0.0007	0.0245	0.0283	0.0006
0.8	0.7	0.8	0.7	0.11	0.18	0.14	0.24	0.2	0.3	0.15	0.15	0.0139	0.0206	0.0001	0.0185	0.0206	0.0005

Power

Se_C	Sp_C	Se_E	Sp_E	$\psi_{D_{true}}$	$\psi_{D_{ass.}}$	$\psi_{ND_{true}}$	$\psi_{ND_{ass.}}$	π_{true}	$\pi_{ass.}$	Non-inferiority margin sensitivity	Non-inferiority margin specificity	Power sensitivity fixed design	Power specificity fixed design	Power overall fixed design	Power sensitivity adaptive design	Power specificitiy adaptive design	Power overall adaptive design
0.8	0.7	0.8	0.7	0.11	0.18	0.14	0.24	0.2	0.3	0.1	0.1	0.8203	0.9996	0.82	0.8168	0.9959	0.8137
0.6	0.7	0.6	0.7	0.11	0.18	0.14	0.24	0.2	0.3	0.1	0.1	0.8238	0.9996	0.8235	0.816	0.9966	0.8138
0.8	0.7	0.8	0.7	0.11	0.18	0.14	0.24	0.2	0.3	0.1	0.1	0.8203	0.9996	0.82	0.8168	0.9959	0.8137
0.8	0.6	0.8	0.6	0.11	0.18	0.14	0.24	0.2	0.3	0.1	0.1	0.8203	0.9998	0.8201	0.816	0.9967	0.8133
0.8	0.8	0.8	0.8	0.11	0.18	0.14	0.24	0.2	0.3	0.1	0.1	0.8203	0.9996	0.82	0.816	0.9965	0.8139
0.8	0.7	0.8	0.7	0.11	0.18	0.14	0.24	0.4	0.5	0.1	0.1	0.9705	0.9879	0.9591	0.8841	0.9355	0.8293
0.8	0.7	0.8	0.7	0.11	0.18	0.14	0.24	0.6	0.7	0.1	0.1	0.9999	0.9848	0.9847	0.9735	0.8477	0.8252
0.8	0.7	0.8	0.7	0.11	0.18	0.14	0.24	0.8	0.9	0.1	0.1	1	0.9989	0.9989	1	0.8282	0.8282
0.8	0.7	0.8	0.7	0.11	0.18	0.14	0.24	0.2	0.1	0.1	0.1	0.9991	1	0.9991	0.8434	0.9997	0.8432
0.8	0.7	0.8	0.7	0.11	0.18	0.14	0.24	0.2	0.4	0.1	0.1	0.7496	0.9985	0.7485	0.8097	0.9947	0.8061
0.8	0.7	0.8	0.7	0.11	0.18	0.14	0.24	0.2	0.5	0.1	0.1	0.7437	0.9982	0.7424	0.8022	0.9927	0.7974
0.8	0.7	0.8	0.7	0.16	0.18	0.14	0.24	0.2	0.3	0.1	0.1	0.6938	0.9996	0.6934	0.8011	0.9985	0.8001
0.8	0.7	0.8	0.7	0.32	0.18	0.14	0.24	0.2	0.3	0.1	0.1	0.4422	0.9996	0.4419	0.7881	1	0.7881
0.8	0.7	0.8	0.7	0.11	0.18	0.21	0.24	0.2	0.3	0.1	0.1	0.8203	0.9932	0.8147	0.8264	0.9855	0.8156
0.8	0.7	0.8	0.7	0.11	0.18	0.42	0.24	0.2	0.3	0.1	0.1	0.8203	0.894	0.7334	0.8763	0.923	0.811
0.8	0.7	0.8	0.7	0.11	0.18	0.14	0.24	0.2	0.3	0.05	0.05	0.8391	0.9999	0.839	0.799	0.9959	0.7967
0.8	0.7	0.8	0.7	0.11	0.18	0.14	0.24	0.2	0.3	0.15	0.15	0.7055	0.9994	0.705	0.8111	0.9969	0.8089

Sample size and bias under the alternative hypothesis

Se_C	Sp_C	Se_E	Sp_E	$\psi_{D_{true}}$	$\psi_{D_{ass.}}$	$\psi_{ND_{true}}$	$\psi_{ND_{ass.}}$	π_{true}	$\pi_{ass.}$	Non-inferiority margin sensitivity	Non-inferiority margin specificity	true sample size	sample size fixed design	sample size interim analysis	sample size adaptive design	$\hat{\pi}$	bias = $\frac{\hat{\pi}-\pi_{true}}{\pi_{true}}$	$\hat{\psi}_D$	bias = $\frac{\hat{\psi}_D-\psi_{D_{true}}}{\psi_{D_{true}}}$	$\hat{\psi}_{ND}$	bias = $\frac{\hat{\psi}_{ND}-\psi_{ND_{true}}}{\psi_{ND_{true}}}$
0.8	0.7	0.8	0.7	0.11	0.18	0.14	0.24	0.2	0.3	0.1	0.1	551	548	158	581	0.1997	-0.0014	0.1114	0.0131	0.1402	0.0014
0.6	0.7	0.6	0.7	0.11	0.18	0.14	0.24	0.2	0.3	0.1	0.1	551	548	158	577	0.1997	-0.0014	0.1105	0.0044	0.1402	0.0014
0.8	0.7	0.8	0.7	0.11	0.18	0.14	0.24	0.2	0.3	0.1	0.1	551	548	158	581	0.1997	-0.0014	0.1114	0.0131	0.1402	0.0014
0.8	0.6	0.8	0.6	0.11	0.18	0.14	0.24	0.2	0.3	0.1	0.1	551	548	158	581	0.1997	-0.0014	0.1114	0.0131	0.1394	-0.0042
0.8	0.8	0.8	0.8	0.11	0.18	0.14	0.24	0.2	0.3	0.1	0.1	551	548	158	581	0.1997	-0.0014	0.1114	0.0131	0.1395	-0.0037
0.8	0.7	0.8	0.7	0.11	0.18	0.14	0.24	0.4	0.5	0.1	0.1	318	465	106	332	0.4	0.0001	0.1107	0.0067	0.1409	0.0062
0.8	0.7	0.8	0.7	0.11	0.18	0.14	0.24	0.6	0.7	0.1	0.1	340	664	158	351	0.5999	-0.0002	0.1109	0.0079	0.14	0.0001
0.8	0.7	0.8	0.7	0.11	0.18	0.14	0.24	0.8	0.9	0.1	0.1	648	1985	473	659	0.8001	0.0001	0.1106	0.0051	0.1405	0.0036
0.8	0.7	0.8	0.7	0.11	0.18	0.14	0.24	0.2	0.1	0.1	0.1	551	1564	473	562	0.1999	-0.0003	0.1107	0.0064	0.1401	0.001
0.8	0.7	0.8	0.7	0.11	0.18	0.14	0.24	0.2	0.4	0.1	0.1	551	471	119	585	0.2	-0.0002	0.1106	0.0051	0.1404	0.0031
0.8	0.7	0.8	0.7	0.11	0.18	0.14	0.24	0.2	0.5	0.1	0.1	551	465	106	591	0.1997	-0.0013	0.1115	0.0134	0.1404	0.0031
0.8	0.7	0.8	0.7	0.16	0.18	0.14	0.24	0.2	0.3	0.1	0.1	714	548	158	747	0.1997	-0.0014	0.1609	0.0053	0.1402	0.0014
0.8	0.7	0.8	0.7	0.32	0.18	0.14	0.24	0.2	0.3	0.1	0.1	1284	548	158	1296	0.1997	-0.0014	0.3127	-0.0229	0.1402	0.0014
0.8	0.7	0.8	0.7	0.11	0.18	0.21	0.24	0.2	0.3	0.1	0.1	556	548	158	590	0.1997	-0.0014	0.1114	0.0131	0.209	-0.0048
0.8	0.7	0.8	0.7	0.11	0.18	0.42	0.24	0.2	0.3	0.1	0.1	632	548	158	664	0.1997	-0.0014	0.1114	0.0131	0.4204	0.001
0.8	0.7	0.8	0.7	0.11	0.18	0.14	0.24	0.2	0.3	0.05	0.05	1893	2066	381	1948	0.1998	-0.001	0.1106	0.0051	0.14	-0.0001
0.8	0.7	0.8	0.7	0.11	0.18	0.14	0.24	0.2	0.3	0.15	0.15	283	261	94	354	0.2003	0.0015	0.1503	0.3663	0.1496	0.0688
